# The impact of mechanical devices for lifting and transferring of patients on low back pain and musculoskeletal injuries in health care personnel—A systematic review and meta‐analysis

**DOI:** 10.1002/1348-9585.12423

**Published:** 2023-09-15

**Authors:** Hans‐Udo Richarz, Arturo Tamayo, Jan Rahmig, Timo Siepmann, Jessica Barlinn

**Affiliations:** ^1^ Division of Health Care Sciences Dresden International University Dresden Germany; ^2^ Winnipeg Regional Health Authority (WRHA), Department of Medicine, Section of Neurology University of Manitoba Winnipeg Manitoba Canada; ^3^ The Max Rady Faculty of Health Sciences, Brandon Regional Health Centre University of Manitoba Winnipeg Manitoba Canada; ^4^ Department of Neurology University Hospital Carl Gustav Carus, Technische Universität Dresden Dresden Germany

**Keywords:** health care workers, low back pain, mechanical patient handling devices, meta‐analysis, musculoskeletal injuries, systematic review

## Abstract

**Objectives:**

Heavy lifting in nursing is highly associated with low back pain (LBP) and musculoskeletal injuries (MSI). We aimed to evaluate the impact of mechanical devices used for patient lifting and transferring on risk of LBP and MSI of health care personnel.

**Methods:**

We conducted a systematic review and meta‐analysis. The literature search was performed during 1st and 12th September 2021 using 17 electronic databases and handsearching of bibliographies of included studies. Twenty studies were included in the qualitative synthesis and eight studies with in total 2087 participants in the meta‐analysis. Dependent on the study design, risk of bias was assessed by Cochrane RoB 2.0, EPOC, and MINORS. We conducted random‐effects meta‐analyses assessing Hedges's g and 95% CI of MSI rate, perceived LBP, and peak compressive spinal load. We calculated prediction intervals and conducted a cost‐benefit analysis (CBA).

**Results:**

All outcomes showed significant, adjusted pooled effect sizes (MSI rate: g = 1.11, 95% CI 0.914–1.299; perceived LBP: g = 1.54, 95% CI −0.016–3.088; peak compressive spinal load: g = 1.04, 95% CI −0.315 to 2.391). True effect sizes in 95% of all comparable populations fell in the following prediction intervals: MSI rate = −1.07‐3.28, perceived LBP = −0.522–3.594, and peak compressive spinal load = −15.49 to 17.57. CBA revealed cost‐benefit ratios of 1.2 and 3.29 between cumulative total savings and investment costs of intervention.

**Conclusions:**

Prediction intervals confirmed strong true effect sizes for MSI rate and perceived LBP in 95% of all comparable populations but not for peak compressive spinal load. Mechanical lifting and transferring devices displayed a favorable cost‐benefit ratio and should be considered for clinical implementation.

## 1 INTRODUCTION

A recent survey by the American Nurses Association (ANA) reported that 51% of nurses suffer from occupational musculoskeletal pain^1^ from which low back pain (LBP) was most at risk,^2^ in particular, when “transferring and moving patients”.^3^ Associated costs with back injuries in the US health care sector are estimated to be US$ 20 billion annually.^4^

Various treatment and management strategies are discussed to avoid the development of these complications including both individual measurements to be initiated by the health care personnel themselves, e.g., weight reduction, diet, physical exercise, or similar, and administrative measurements to be set up by the health facilities, e.g., training in manual handling, setup of lift teams, and ergonomic multicomponent programs.^2^ Particularly, the effectiveness of mechanical patient handling devices for the back health of nurses,^4^ but also their drawbacks in daily practical use, e.g., lack of availability and use, are discussed.^5^ However, there is some evidence^7^ that the use of mechanical patient handling devices may reduce both MSI and costs associated with workers’ compensation claims.^1^, ^4^

Apart from the recently reported results of unconvincing evidence related to the effectiveness of small aids, e.g., sliding sheets, walking belts, etc. on musculoskeletal outcomes,^6^ the reliability of results for both mechanical,^8^ and other types of interventions^7^ are questioned particularly due to the weak methodological quality of the studies.^7^, ^8^, ^9^ In most studies, the impact of mechanical handling devices is not examined separately but as part of a multicomponent strategy or combined with small aids making it impossible to attribute possible beneficial effects to the use of mechanical patient handling devices.^8^

Therefore, the objective of this systematic review and meta‐analysis was to primarily evaluate the impact of mechanical devices when used for patient lifting and transferring tasks on both LBP and MSI of health care personnel. Because some handling tasks, e.g., repositioning, reportedly have different risks of causing MSI including claim costs^10^, ^11^ we focused this review specifically on lifting and transferring tasks. As mechanical patient handling devices we considered all mobile and fixed technically patient or resident handling equipment that is in general electrically driven via wall socket or battery, e.g., patient ceiling‐ or floor lifts or similar. Based on the assumption that low back load is strongly discussed as an independent factor contributing to LBP,^12^ we also focused on biomechanical studies measuring mechanical load of low back as “quasi‐objective” LBP outcome.

## 2 MATERIALS AND METHODS

The systematic review and meta‐analysis were based on the recommendations of Cochrane Handbook^13^ and PRISMA guidelines (Appendix A).^14^

Details of the systematic review were registered on PROSPERO International Prospective Register of Systematic Reviews (No of registration: CRD42021297165).

### 2.1 Literature search and study selection

**FIGURE 1:**
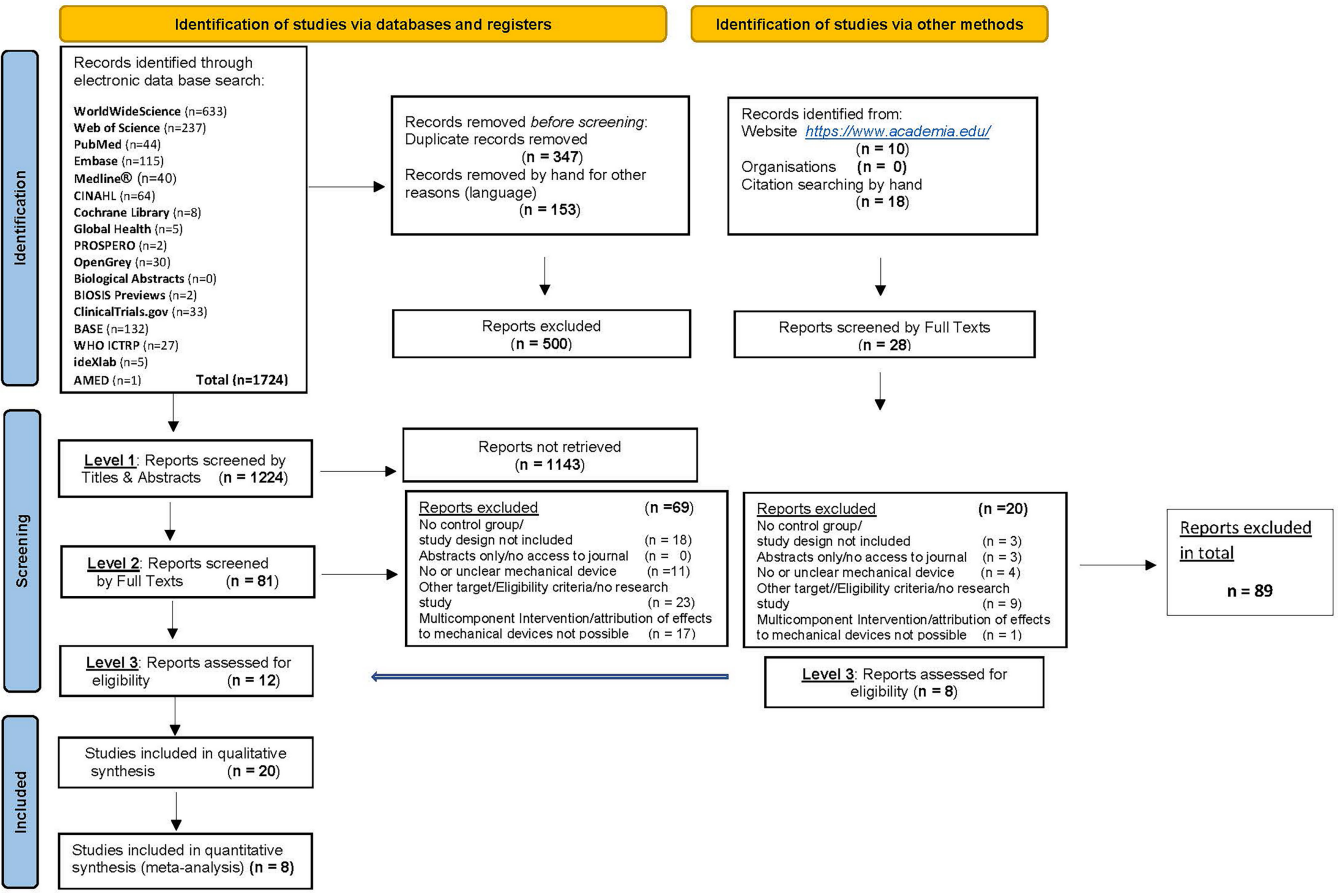
“Flow diagram of literature search”.

The literature search was conducted between 1st September and 12th September 2021 via electronic databases to be seen from Figure 1 and via EThOS (dissertations & theses) on their website https://ethos.bl.uk. The literature search was not limited to a certain period or language restrictions.

Based on the research question and a pre‐defined search strategy, the basic concepts for the electronic literature search were nursing/health care workers, LBP/‐injuries, MSI, and mechanical patient lifts/assistive devices, appropriately exploded by using specific indexed vocabulary (MeSH terms and free text) as well as applying field tags, Boolean operators, parentheses, and quotation marks. The search strategy, using the example of PubMed, is shown in Appendix B. The results were exported to the literature management system Endnote 20; duplicates were eliminated.

The reference lists of the full‐text papers included were hand‐searched for any other publications that could not be identified by electronic search. Further records were identified from the website https://www.academia.edu/. Two reviewers agreed on a standard approach for screening. Then, the records were firstly screened in terms of titles and abstracts by one reviewer and in a second step in terms of full texts by two reviewers. Discrepancies were resolved by a third reviewer.

### 2.2 Eligibility criteria

The research question and eligibility criteria were compiled using the ECLIPSE‐framework presented in Table 1.

**TABLE 1:** ECLIPSE_eligibility criteria.

Research question	Do mechanical patient handling devices have an impact on LBP and MSI among nurses and other HCWs?
ECLIPSE	Eligibility Criteria
Expectation	Reduction of work‐related LBP and MSI among nurses and other health care workers (HCWs) being directly involved in patient/resident handling tasks. The basic question is whether this objective may be reached by the exclusive use of mechanical devices.
Client Group	In particular, nurses of any kind but also HCWs in a broader sense suffering from LBP (specific or unspecific, acute or chronic) and/or MSI, and regularly performing patient/resident lifting and transferring tasks.
Location	Any kind of stationary inpatient (hospitals) or outpatient care, home care, elderly care, foster homes, or similar, where regular patient/resident handling tasks are to be performed.
Impact	Effectiveness of mechanical patient/resident handling devices (e.g., ceiling lifts, floor lifts, etc.) on occupational LBP or MSI of nurses and other HCWs. The effectiveness will be measured by the decrease of LBP or MSI via the use of mechanical equipment in patient/resident lifting and transferring (intervention/exposure) compared to exclusive manual (with or without small aids) patient/resident handling in the same nurse‐ or HCW‐setting (control).
Professionals	Involved in the services to be provided being, as the case may be, the necessary availability of mechanical equipment (availability), its adequate storage, accessibility, maintenance, and provision of awareness and training as well are the responsible health care facilities and insurance companies. The nurses or other HCWs as “Client Group” are involved in this process by regular and appropriate use of the mechanical patient/resident handling equipment.
Service	Patient handling services are provided in any kind of stationary inpatient (hospitals) or outpatient care, home care, elderly care, foster homes, or similar.
Study Design	Randomized controlled trials (RCTs), controlled before‐after studies, controlled observational studies (cohort‐ and cross‐sectional studies), and biomechanical studies on mechanical spinal load (L4/L5/S1) as well.

We excluded narrative and systematic reviews, editorials, expert opinions, commentaries, case series, case reports, and, in general, all papers not entailing a research study. Excluded were also studies reviewing multicomponent (patient handling) programs, the mechanical patient handling devices may be part of, if the impact of one or more mechanical patient handling device(s) is/are not tested/measured separately in the study (as part of the multicomponent program) and therefore the impact cannot be separately evaluated. We also excluded studies if evaluation could not be separately done regarding the type of handling task of interest, i.e., lifting and transferring tasks (to be distinguished from other handling tasks, e.g., patient repositioning).

### 2.3 Data extraction

Data extraction was independently done by two reviewers using a standardized spreadsheet with the characteristics: (1) authors and year of publication of the study, (2) study design, (3) sample size, (4) population of interest, (5) study duration, (6) intervention (exposure)/comparator, (7) outcome (impact), and (8) results. With regard to the cost‐benefit analysis (CBA) the following data were extracted to the extent they were available from the included studies: (i) working days lost, (ii) lost workday injury, (iii) total claim compensation costs (musculoskeletal injuries), (iv) investment costs for intervention, (v) savings (direct and indirect). In case of any missing or unclear information, we excluded the study from the quantitative analysis.

### 2.4 Risk of bias—Quality assessment methods

With regard to the methodological quality rating of the studies, the following tools were used: For the randomized controlled study of Yassi et al. (2001)^15^ and the biomechanical sub‐study related hereto from Daynard et al. (2001),^16^ the rating was assessed by the Cochrane RoB 2.0 tool.^17^ With regard to the non‐randomized study designs of both before‐after studies and interrupted time series studies, the quality rating was done by means of the EPOC RoB tool.^18^ For the biomechanical studies, the MINORS tool was used.^19^ The quality ratings were separately done by two reviewers. Any discrepancies were resolved by a third reviewer.

For the evaluation of the certainty of pooled effect size estimates, the approach of GRADE (Grading of Recommendations, Assessment, Development, and Evaluations) as stated in the GRADE‐Handbook^20^, ^21^ was used.

### 2.5 Data synthesis

Separate (quantitative) random‐effects meta‐analyses were conducted to evaluate the outcomes perceived LBP, peak compressive spinal load on L4/L5/S1, and rate of MSI. A narrative review was done for the resting outcomes as far as relevant.

In the studies that evaluated MSI rate and peak compressive spinal load, the results were tested and assessed by the researchers in one (matched) group of subjects (with or without a control group), respectively. Thus, the within‐group effect sizes and their 95% confidence interval before and after the intervention were calculated. Regarding perceived LBP, the results were tested and assessed by the researchers in an experimental and a control group, thus calculating the between‐group effect sizes and their 95% confidence interval after implementation of the intervention. Whereas the data for perceived LBP (mean/standard deviation) could be directly used as input for the meta‐analysis software program, the necessary input data (studies’ mean difference and SD of difference) for calculating the pooled effect sizes of MSI rate and peak compressive spinal load had to be pre‐calculated by conducting two‐tailed paired *t*‐tests accordingly. We qualified the outcome MSI rate as a continuous variable for two reasons: firstly, due to its infinite nature (being a continuum coming from a real number line)^22^ and secondly considering the fact that also a fraction of the MSI rate, e.g., the half of a rate of 4.6, does make sense.^22^ Before conducting these parametric paired t‐tests, the data were tested for normal distribution by using histograms, Shapiro‐Wilk tests, or, if so, using the principle of the Central Limited Theorem (CLT). As effect size for all three meta‐analyses, Hedges's g was used instead of Cohen's d (standardized difference of means) in order to avoid a bias which may overestimate the effect size due to the (most) small sample sizes of the studies included.

With regard to the reviewed outcomes, separate exploratory meta‐analyses were performed. Only studies that were comparable in particular regarding the intervention and the outcome measurements were included in the meta‐analyses. Due to the fact that the studies included in each of the meta‐analyses came from a universe of populations (and not from the same population), the random‐effects model was used in each of the quantitative calculations. The between‐studies variance (T^2^) was estimated with the DerSimonian and Laird method. Because the needed pre‐post correlation value was not indicated in the studies, we assumed a—conservative—imputed *r*‐value of .5^23^ for each of the studies when indicating the input data for the meta‐analyses. Heterogeneity was assessed by using an *I* ^2^‐statistics (as proportion) considering low statistical heterogeneity when *I* ^2^ was below 40%.^24^ Additional calculations of corresponding prediction intervals were conducted in order to indicate where the true effects of the intervention can be expected for 95% of comparable populations in future studies,^25^ meaning how much the pooled effect size varies across the studies.^26^

A possible publication bias was evaluated by a funnel plot and by Egger's regression test as well. A sensitivity analysis was conducted using the trim‐and‐fill method.^27^ Both the observed and imputed studies were used to compute an adjusted estimate of an unbiased effect size. A meta‐regression intended to test the influence of study moderators, e.g., study period, worker's rate, age, sex, etc., on the point estimates, could not be conducted in any of the three meta‐analyses due to the small number of studies.

A cost‐benefit analysis (CBA) was conducted on the basis of available and comparable study data. The data of two studies could be included in the analysis and the resulting cost‐benefit ratios including payback periods were graphically displayed by means of histograms.

The data were analyzed using software programs of G*Power 3.1.9.2 (Düsseldorf University), STATA 17 (StataCorp LLC.), Comprehensive Meta‐Analysis (Biostat Inc.), and the online “Prediction Interval Calculator for Random Effects Meta‐analysis” (https://medstats.github.io/ranefpredict.html).

## 3 RESULTS

### 3.1 Study selection and study characteristics

The electronic search retrieved 1724 results that we exported to Endnote 20. Then, we electronically removed 347 duplicate records and 153 records for language reasons, because we restricted the records to studies in English, German, and Italian.

From 15th September 2021 until 29th October 2021, a total of 1224 electronically identified records were screened by one reviewer in a first step in terms of titles and abstracts. In a second step from 29th October until 04th December 2021, a total of 81 full‐text reports were screened independently by two reviewers against the eligibility criteria. The reference lists of the full text reports were hand‐searched for publications that could not be identified by electronic search. Further records were identified from the website https://www.academia.edu/. These additional 28 full‐text reports identified were likewise screened against the eligibility criteria. Discrepancies between the reviewers were resolved by a third reviewer. In total, 89 studies were excluded for reasons (Appendix C). Twenty studies could be included in the systematic review^10^, ^11^, ^15^, ^16^, ^26^, ^27^, ^28^, ^29^, ^30^, ^31^, ^32^, ^33^, ^34^, ^35^, ^36^, ^37^, ^38^, ^39^, ^40^, ^41^ and eight studies in the meta‐analysis.^16^, ^26^, ^32^, ^35^, ^36^, ^39^, ^40^, ^41^ A flow diagram of the literature that was searched and evaluated is shown in Figure 1.

Four of the 20 studies included for qualitative analysis were before‐after studies with a separate control group each, seven studies were interrupted time series studies, and one study was a randomized controlled trial (RCT). Further eight studies, one of them being a sub‐study of the only RCT, were biomechanical studies.

Study characteristics and risk‐of‐bias score of each study included in the systematic review are shown in Tables A1 and A2 of Appendix D.

### 3.2 Effects on low back pain condition and musculoskeletal injuries

We identified as outcomes LBP prevalence in one study,^36^ LBP disability in one study,^15^ perceived/self‐rated LBP in three studies,^15^, ^37^, ^38^ MSI as rate in seven studies,^15^, ^39^, ^40^, ^41^, ^42^, ^43^ MSI in absolute case numbers in 12 studies,^10^, ^11^, ^15^, ^35^, ^36^, ^37^, ^38^, ^39^, ^40^, ^41^, ^42^, ^43^ and peak‐, cumulative compressive‐, and shear spinal load in eight studies.^16^, ^28^, ^29^, ^30^, ^31^, ^32^, ^33^, ^34^

Due to the heterogeneity of outcomes and the small number of studies a meta‐analysis could be conducted on perceived LBP (two studies), peak compressive spinal load (three studies), and MSI rate (three studies) only. However, the results on outcomes that could not be quantitatively analyzed were narratively reported as well.

All studies reporting absolute numbers of MSI as well as some studies reporting MSI rates could not be considered because they did not provide the number of workers at risk. This concerned also the only RCT of Yassi et al. (2001) that we could identify.^15^ Furthermore, this RCT does not differentiate between mechanical patient handling devices and manually used aids (e.g., slide sheets), meaning that the study's intervention Arm C (“no strenuous” lifting program) consisted of both kinds of patient handling methods not reflecting, however, our specific intervention of interest. Nevertheless, the before‐after results from Yassi et al.'s intervention Arm C showed a slight decrease for both MSI in absolute numbers (from 21 cases 1 year before intervention to 17 cases 1 year after intervention) and MSI rate (from 8.2 one year before intervention to 6.1 one year after intervention), resulting in a moderate effect size we calculated on the basis of the foregoing data with Hedges's g = 0.493 (assuming *ß* = .8, *ɑ* = .05, *r* = .5). The outcomes perceived LBP (baseline = pre: 26.5 ± 28.1; 1 year = post: 24.2 ± 25.4) and LBP (Oswestry) disability score (baseline = pre: 5.7 ± 8.0; 1 year = post: 5.4 ± 7.6) as tested by Yassi et al. by means of a (within) before‐after comparison in Arm C showed no significant effects (Hedges's g = −0.0084 and Hedges's g = 0.0036) without indication of reasons therefor by the authors of the study. Knibbe et al. (1999)^36^ reported a within‐decrease in 12‐month back pain prevalence (pre: 74%/post: 64%) in a controlled before‐after study not indicating, however, the corresponding effect size of this decrease. This effect size was calculated and reported by Hegewald et al. (2018)^8^ in their meta‐analysis with RR (random) 0.97 (95% CI: 0.83 to 1.15), therefore reaching no statistical significance.^8^

More specific information on the achieved results in our meta‐analyses conducted:

#### 3.2.1 MSI rate

We conducted a random‐effects model meta‐analysis of three comparable studies (a total of 1918 participants) to estimate a pooled effect size by means of a pre‐post comparison within the same (matched) group of subjects.

**FIGURE 2:**
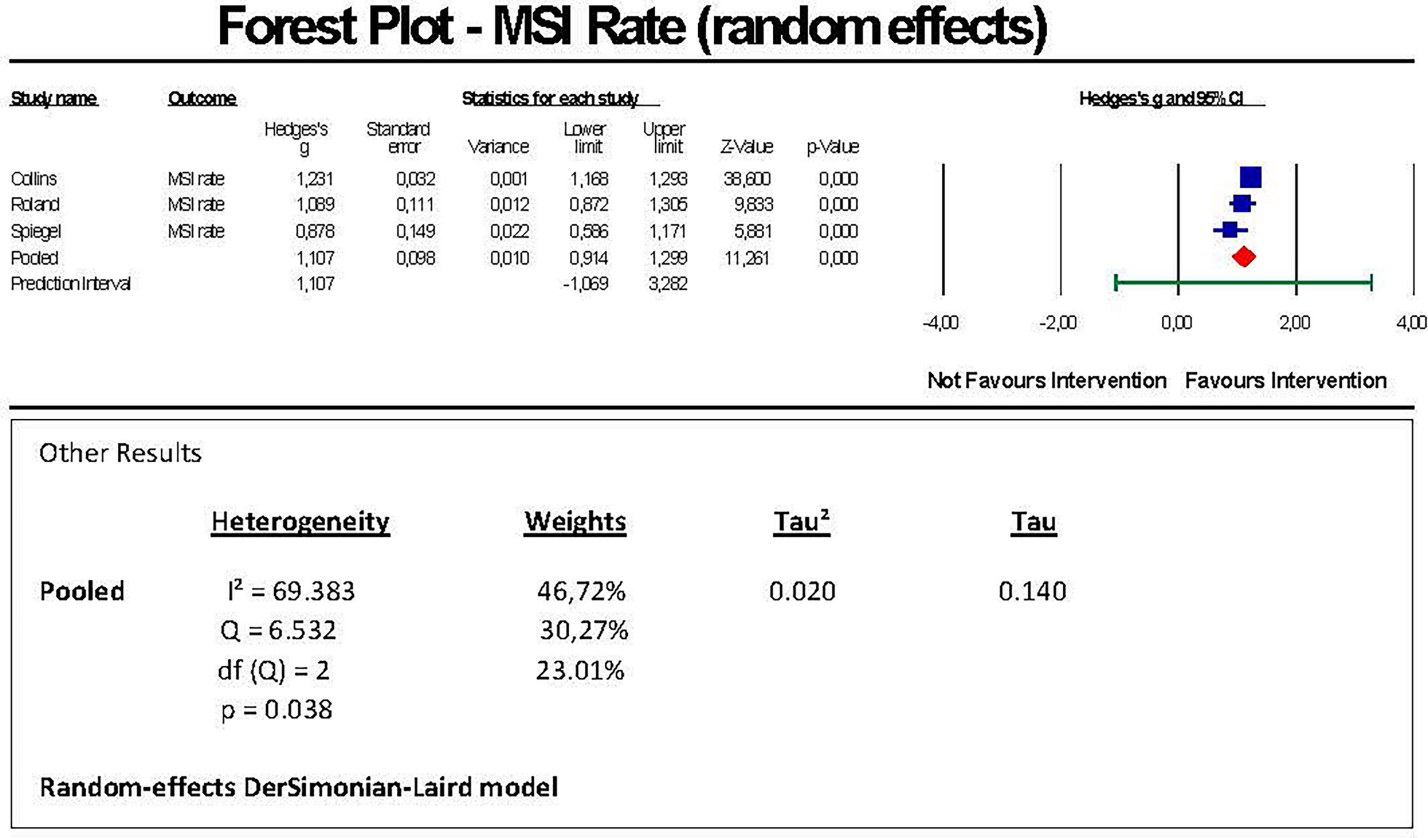
Forest plot MSI rate.

We found a significant (unadjusted) pooled effect size of Hedges's g = 1.107 with a 95% confidence interval of 0.914 to 1.299. The forest plot showed individual Hedges's g effect sizes of the studies included in the range of 0.878 and 1.231 (Figure 2).

The z‐value for testing the null hypothesis, i.e., the mean Hedges's g is 0, was 11.261 with a corresponding *P*‐value of <.001. Thus, we could reject the null hypothesis that the impact of the intervention is the same before and after the intervention concluding that the intervention is favorable across the studies. Heterogeneity showed a *p*‐value of .038 rejecting the null hypothesis that there will be no heterogeneity between the studies. The distribution of weight of studies was 46.72%/30.27%/23.01%. A funnel plot (Appendix E, Table A1) showed no publication bias which was confirmed by Egger's regression test showing a significant result (*P* = .15840). A sensitivity analysis by means of the Duval and Tweedie's trim‐and‐fill method was conducted demonstrating that due to the non‐existent publication bias, the pooled effect size kept unchanged (Hedges's g = 1.10653 (95% CI = 0.91394 to 1.29913). *I* ^2^‐statistics showed a proportion of 69.38% of the observed variance that reflects differences in the true effect sizes, that is, not due to sampling error. The Q‐value was 6.532 with 2 degrees of freedom (df) and *P* = .038. To evaluate variation of the pooled effect size across the studies, the prediction interval was calculated (1.07 to 3.28) as shown graphically in Appendix F, Figure A1. Tau‐squared (variance of true effect sizes) was 0.020. Tau (standard deviation of true effect sizes) was 0.140.

#### 3.2.2 LBP perceived

**FIGURE 3:**
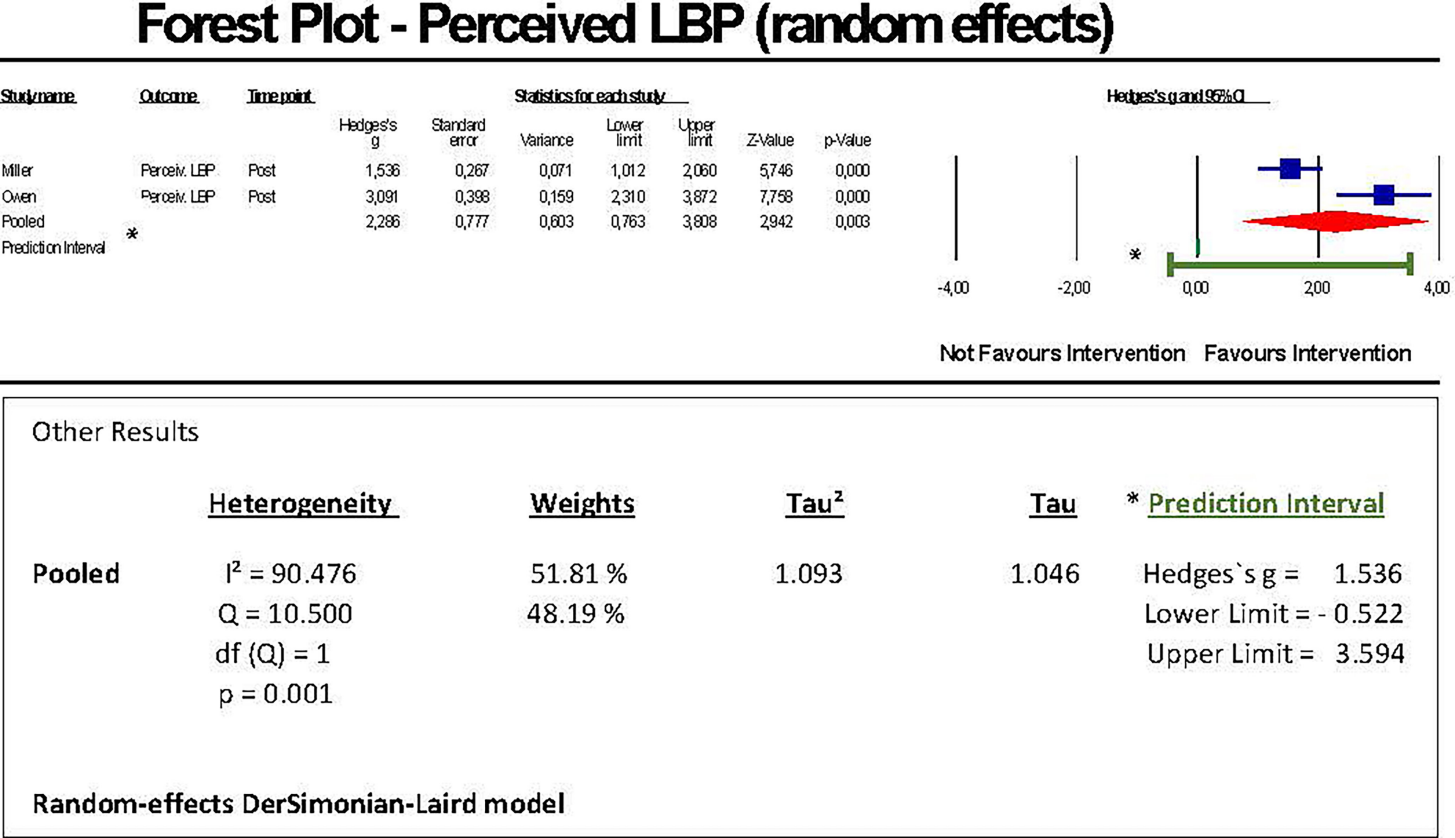
Forest plot perceived LBP.

We conducted a random‐effects model meta‐analysis of two comparable studies (a total of 131 participants) to estimate a pooled effect size on the outcome by means of post‐intervention comparisons between an experimental group and a separated control group. The outcome data (mean/standard deviation) of the studies could be directly used for running a meta‐analysis. We found a significant (unadjusted) pooled effect size of Hedges's g = 2.286 with a 95% confidence interval of 0.763 to 3.808. The forest plot showed individual Hedges's g effect sizes of the two studies in the range of 1.54 and 3.09 (Figure 3).

The z‐value for testing the null‐hypothesis, i.e., that the mean Hedges's g is 0, was 2.94 with a corresponding *p*‐value of .0033. Thus, we could reject the null hypothesis that the impact of the intervention after its implementation is the same between experimental and control groups concluding that the intervention is favorable across the studies. Heterogeneity showed a *p*‐value of .0012 rejecting the null hypothesis that there will be no heterogeneity between the studies. The distribution of weight of studies was 51.8%/48.2%. A publication bias could be seen when displaying a funnel plot (Appendix E, Table A2). A publication bias was not indicated by Egger's regression test (*P* = .0576), a test that might be, however, possibly less powerful if—as here—a few effect sizes only can be pooled.^56^ To additionally reveal if this asymmetry shown by the funnel plot was due to publication bias, a sensitivity analysis by means of Duval and Tweedie's trim‐and‐fill method was conducted demonstrating that due to the existent publication bias, the pooled effect size changed from Hedges's g = 2.286 (95% CI 0.767–3.863) to Hedges's g = 1.536 (95% CI = −0.016‐3.088). *I* ^2^‐statistics showed a proportion of 90.476% of the variance of true effects, i.e., not due to sampling error. The Q‐value was 10.500 with 1 degree of freedom (df) and *P* = .001. To evaluate the variation of the mean effect size across the studies, the prediction interval was calculated (−0.522 to 3.594) as shown graphically in Appendix F, Figure A2. Tau‐squared was 1.093. Tau was 1.046.

#### 3.2.3 Peak compressive spinal load

**FIGURE 4:**
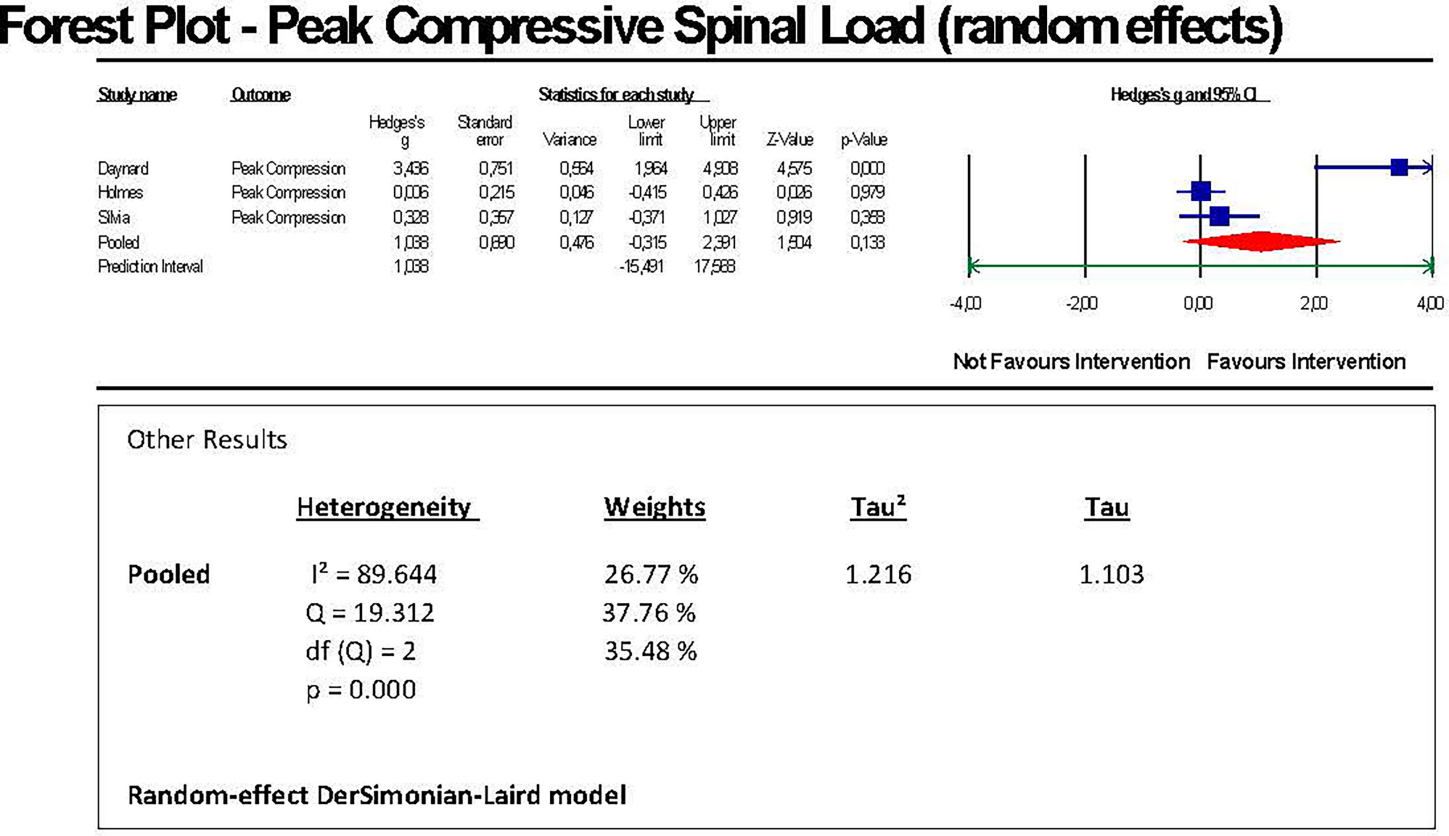
Forest plot LBP peak compressive spinal load.

We conducted a random‐effects model meta‐analysis of three comparable studies (a total of 38 participants) to estimate a pooled effect size by means of pre‐post comparisons within the same (matched) group of subjects. In order to get appropriate input data (mean difference and standard deviation of difference) for running a meta‐analysis, we conducted in the first step a two‐tailed paired *t*‐test using mean differences of Newton as indicated in the studies. The outcome was assessed either by the unit Newton^16^, ^28^ or—in the study of Silvia et al. (2002)^34^—by the unit Pounds (lbs). Therefore, we converted Pounds into Newton by the factor 4.445 in order to harmonize the basis of statistical calculation. With regard to the mean/standard error (S.E.) used by Daynard et al. (2001),^16^ we converted for reasons of harmonization the unit S.E. into SD (standard deviation) by using the formula SD = S.E.* square root of N. Because the needed pre‐post correlation value was not indicated in the studies, we assumed a—conservative—imputed *r*‐value of .5 for each of the three studies when indicating the input data for the meta‐analysis. We found a significant (unadjusted) pooled effect size of Hedges's g = 1.038 with a 95% confidence interval of −0.315 to 2.391. The forest plot showed individual Hedges's g effect sizes of the three studies in the range of 0.328 and 3.436 (Figure 4).

The z‐value for testing the null hypothesis, i.e., that the mean Hedges's g is 0, was 1.504 with a corresponding *p*‐value of .133. Thus, we could not reject the null hypothesis that the impact of the intervention is the same before and after its implementation concluding that the intervention seemed to be statistically not significant across the studies. Heterogeneity showed a *p*‐value of <.001 rejecting the null hypothesis that there will be no heterogeneity between the studies. The distribution of weight of studies was 51.8%/48.2%. A funnel plot (Appendix E, Table A3) did not reveal a publication bias which was confirmed by the Egger's regression test showing a significant result (*P* = .19689). A sensitivity analysis was conducted demonstrating that due to the non‐existent publication bias, the pooled effect size was kept unchanged (Hedges's g = 1.03811; 95% CI = −0.31479 to 2.39101). Heterogeneity, assessed by the *I* ^2^‐ statistics, showed a proportion of 89 644% of the variance of true effects, i.e., not due to sampling error. The Q‐value was 19.312 with 2 degrees of freedom (df) and *P* = .000. The prediction interval was calculated with −15.49 to 17.57 as shown graphically in Appendix F, Figure A3. Tau‐squared was 1.216. Tau was 1.103.

### 3.3 Methodological quality assessment

#### 3.3.1 Risk of bias

The quality ratings of the studies included are shown in separate table formats (Table A1 to Table A5) for each used risk bias tool in Appendix G.

Thirteen studies showed a high risk of bias including the RCT and eight biomechanical studies. Of low risk of bias were six interrupted time series studies and one before‐after study.

#### 3.3.2 Certainty of evidence

The certainty of evidence for the outcomes included in the meta‐analyses (Table 2) was evaluated as per the GRADE approach as described in the Methods section.

**TABLE 2:** GRADE Approach; Quality of the Bodies of Evidence ‐ Summary of Findings.

GRADE‐Approach—Summary of Findings	The impact of mechanical devices used for patient lifting and transferring on low back pain and musculoskeletal injuries of nurses and other health care workers					
Outcomes	Anticipated *Absolute* Effects (95% CI)^a^, Random^b^ Adjusted^c^		Anticipated *Relative* Effects (95% CI)^a^, Random^b^ Adjusted^c^			
	Effect size Hedge's g CI 95%		Effect size Hedge's g CI 95%		No of participants (Studies)	Certainty of the evidence (GRADE)
Musculoskeletal injury rate *(Rate)*	Not computable due to unreported number of workers at risk		1.10653	0.91394*–*1.29913	1918 (3 studies)	LOW 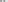
Perceived low back pain *(mean/SD)*	1.536	−0.016*–*3.088	**—**	—	131 (2 studies)	VERY LOW 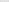
Peak compressive spinal load *(Newton)*	1.03811	−0.31479*–*2.39101	—	—	38 (3 studies)	VERY LOW 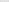

^a^ CI = Confidence Interval.

^b^ Random = random effects Der Simonian and Laird model.

^c^ Adjusted = adjusted values based on sensitivity analyses done by means of Duval & Tweedie's trim‐and‐fill method.

For each of the outcomes, the quantitative evaluation started from low certainty since all studies included were non‐randomized (quasi‐experimental or biomechanical) studies. With regard to the outcome MSI rate, neither a down‐rating nor an up‐rating was justified. Nevertheless, due to the low certainty with regard to the non‐randomized study design the overall certainty of evidence in this case was low. Concerning the outcomes perceived low back pain and peak compressive spinal load down‐ratings due to risk of bias (several methodological limitations), inconsistency of effects (the *I* ^2^‐statistics ranged between 89.6% and 90.5%), imprecision (wide confidence interval), indirectness (the latter only with regard to the biomechanical outcome), and publication bias were necessary. There were no reasons for up‐ratings. Therefore, relating to these two outcomes the overall certainty of evidence turned out to be very low.

### 3.4 Cost‐benefit analysis

The use of mechanical lifting and transferring of patients as intervention of interest showed that MSI could be reduced effectively (Hedges's g = 1.11; 95% = CI 0.913 to 1.299). A Cost‐Benefit analysis (CBA) could be conducted on two studies,^11^, ^43^ reviewing whether potential net benefits (benefits minus costs)^44^ resulting from the intervention exceed the costs of its implementation. The shown reductions in MSI served as the required “welfare changes attributable to the intervention”.^45^ Both costs and benefits were expressed in monetary units (Ca$). The total investment cost of implementation of the intervention (staff training included) and the total savings (direct and indirect savings) were reviewed for a 12‐month post‐period after intervention in both studies. Whereas the direct savings could be directly extracted from the included studies themselves, the indirect savings were conservatively estimated to be double of direct cost savings.^46^ In order to determine the payback period of the investment, a life span of 12 years for the mechanical equipment of interest (in both studies being ceiling lifts) was assumed.^11^, ^43^ For calculating break‐even/payback periods, cost‐benefit ratio histograms were generated demonstrating the cumulative development of both costs and total savings during 1 year following intervention each over the assumed equipment's life span of 12 years.

The histograms on the ratio between the cumulated investment and the cumulated direct/indirect savings of both studies (Appendix H, Table A1, and Table A2) showed an amortization of total investment within the equipment's life span each. However, both the break‐even point and the payback period (taking into consideration direct and indirect savings) were different: in the study of Engst et al. (2005)^11^ (Appendix H/Table A1), the breakeven took place in the 10th year (cost‐benefit ratio = 1.22), whereas in the study of Spiegel et al. (2002)^43^ (Appendix H/Table A2), the breakeven was reached much more earlier and the payback period amounted to 3 to 4 years (cost‐benefit ratio = 3.92).

## 4 DISCUSSION

### 4.1 Comparison with previous results of systematic reviews and meta‐analyses

Our results showed meaningful pooled effect sizes for all analyzed outcomes. The prediction intervals confirmed clinically favorable true effect sizes across the studies in 95% of all comparable populations for MSI rate and perceived LBP only but not for peak compressive spinal load. Mechanical lifting and transferring equipment displayed a favorable cost‐benefit ratio.

Recent systematic reviews on interventions aimed at reducing LBP and MSI focused either on small, not mechanical aids supporting manual patient handling tasks only^6^ or testing a narrower population^7^ or a broader population of interest^7^ or on other interventions, e.g., manual handling or exercise training^47^ or on multicomponent interventions^48^, ^49^ without, however, clearly differentiating between manual based and mechanically based interventions.^15^, ^47^, ^50^, ^51^, ^52^

Richardson et al. (2018)^7^ systematically reviewed studies testing—inter alia—patient lift systems on MSI of registered nurses, including eight studies fulfilling the inclusion criteria. The authors reported that there was no evidence that the “patient lift systems are effective at preventing injuries or pain among [registered] nurses”.^7^ The qualitative ratings were reported as continuously weak. Despite the availability of eight studies a meta‐analysis was not conducted. Hegewald et al. (2018)^8^ conducted a systematic review and meta‐analysis on “technical aids”, which are defined in terms of mechanical patient handling devices, on “musculoskeletal complaints of Health Care Workers”.^8^ The authors, using partly other inclusion criteria as used herein, included 11 studies in their systematic review (1 RCT and 10 controlled before‐after studies), but conducted corresponding meta‐analyses (with four and two studies, respectively) on the outcomes of back pain prevalence (RR) and acute MSI (RR) only. The only RCT from Yassi et al. (2001)^15^ was excluded by the authors from quantitative analysis for the same reason we excluded this study: a clear differentiation between mechanical handling devices from manual aids was not possible. Whereas Hegewald et al. (2018)^8^ reported a decrease in back pain prevalence (based on two studies) being not statistically significant (RR 0.78; 95% CI: 0.44 to 1.37), the results on the pooled effect size for acute MSI (RR: 0.78; 95% CI: 0.68 to 0.90), however, favored the intervention (mechanical equipment) concluding that musculoskeletal complaints can be prevented.^8^ Overall, the authors declare the pooled effects as not convincing due to the limited methodological quality of the studies included and the small number of studies that could be considered in the meta‐analysis.^8^

### 4.2 Evaluation of results

We pinpointed the low until very low methodological quality of the studies reviewed herein. Although we focused besides RCTs on controlled before‐after‐, observational‐, and interrupted time series‐studies, literature search revealed only a few comprehensive studies. The reason why we could not include one of the partly high‐quality observational studies in our systematic review was attributed to our strict eligibility criteria of the exclusive use of mechanical patient handling devices for patient lifting and transferring tasks and the comparability of the outcomes of interest. With regard to the worldwide practical importance of the large number of health care personnel with occupational LBP/MSI and the MSI compensation claims linked herewith,^4^ we expected more studies with clinical, ideally objective, medically diagnosed outcomes but surprisingly we found relatively small number of studies with mainly subjective LBP outcomes only. The most relevant studies we could include evaluated the outcome MSI based on administrative claims data, bearing the risk that these data might not be complete in return leading to a potential selection bias.^8^

In total, we could include eight studies conducting three separate quantitative analyses. The results primarily demonstrated a substantial, adjusted pooled effect size for each outcome a meta‐analysis was conducted for (between Hedges's g 1.04 and 1.54). Unlike the 95% confidence interval of the MSI outcome (95% CI 0.91‐1.30), the 95% confidence intervals of the perceived LBP outcome (95% CI −0.016 to 3.088) and of the peak compressive spinal load‐outcome (95% CI −0.32 to 2.39) as well showed a relatively larger imprecisement with regard to the results of the observed studies by partly falling also into the no‐(favorable) effect (minus)‐range left of the zero. The main reason for these imprecise 95% confidence intervals of all three outcomes may particularly be, but not limited to, the small number of studies and the small sample size of most of the studies included.

A clear—clinically important—tendency of the intervention's favorability for future studies with comparable populations was illustrated by calculating prediction intervals.

Firstly, the MSI prediction interval of −1.07 to 3.28 confirmed the outcome's substantial favorable pooled effect size. Secondly, the MSI prediction interval demonstrated more than 3‐fold favorable effects than no‐favorable effects with regard to 95% of all comparable populations. This result could not be reflected by the outcome's pooled effect size and its 95% confidence interval standing alone.

The perceived (subjective) LBP outcome showed a similar, even slightly higher, substantial pooled effect size (g = 1.536). Its prediction interval was wider (0.522 to 3.594) than the MSI prediction interval, therefore showing a slightly broader spread of the true effect size across the studies. The values were predominantly located in the favorable true effect area, showing more than 5‐fold favorable effects than no‐favorable effects in 95% of all comparable populations.

The pooled effect size of the peak compressive spinal load outcome showed a substantial effect size too (g = 1.04), its prediction interval, however, was very large (−15.49 to 17.57), crossing substantially the no‐true effect range of zero. The true effect size of the peak compressive spinal load outcome was nearly equally distributed between the favorable true effect area and the no‐favorable true effect area in 95% of all comparable populations.

Thus, the meta‐analyses of the two related LBP outcomes (perceived LBP and peak compressive spinal load) seem to diverge in what concerns the distribution of favorable true effects and of no‐favorable true effects across the studies. Nevertheless, there is a slight tendency also with regard to the peak compressive spinal load outcome to show clinically higher favorable true effects than no‐favorable true effects across the studies. This observation reveals that the strength of the clinical (subjective) outcome of perceived LBP may be considered as confirmed by the similarly large pooled effect size of the biomechanical outcome (peak compressive spinal load), being deemed an independent factor for clinical LBP condition^3^ only. No congruence, however, could be seen with regard to the clinically relevant dispersion of the outcomes' true effect sizes across the studies in 95% of all comparable populations. The reasons for this strong divergence with regard to the prediction intervals of the two related LBP outcomes are not clear. In both cases, sample sizes were small (perceived LBP) to very small (peak compressive spinal load).

Overall, the shown values of prediction intervals may be seen as “clinically relevant thresholds”^25^ for future, comparable studies indicating the boundaries between no (true) effects, beneficial‐ or non‐beneficial (true) effects of an intervention in absolute numbers. However, this conclusion should be verified by larger, preferably controlled high‐quality observational studies.

Reasons for possibly not reliable results with regard to LBP outcomes were discussed by Gold et al. (2017).^53^ Based on their cohort study results on the effectiveness of a “safe resident handling programme (SRHP)”, implemented by US‐nursing facilities, they report a delayed time effect of the intervention on a significant improvement of LBP symptoms, meaning that the health care personnel tested “may not experience reduced LBP risk until 2‐6 years post intervention”.^53^ In light of these foregoing reports, the study periods of the studies included in our meta‐analysis on the perceived LBP outcome (12 months^37^ and 18 months^38^) might be too short in order to achieve reliable clinical responses. Also, Hinton et al. (2009)^54^ and Burdorf et al. (2013)^9^ had already previously referred to such possible delayed responses on LBP occurrence after implementation of mechanical patient lifts.

The observed substantial decrease of the MSI outcome could be confirmed by the cost‐benefit analysis (CBA) conducted herein. Focusing on the total costs, meaning both direct costs (workers' claim compensation payments) and indirect costs (to be additionally born by the health facility), the latter entailing a much more wider range of costs, e.g., maintenance/operating/recruiting and training charges,^11^ our analysis showed that the full capital investment amount could be recovered within a 12‐years life span of the implemented ceiling lifts. Both studies reviewed showed a favorable cost‐benefit ratio of 1.2^11^ and of 3.92,^43^ respectively, demonstrating that positive impacts of even expensive ergonomic mechanical devices on clinical/public health outcomes on the one side and investments to be done on the other part are not mutually exclusive.

### 4.3 Limitations

This systematic review and meta‐analysis are subject to certain limitations.

Firstly, besides the pooled effect sizes, the prediction intervals may be imprecise because all meta‐analyses conducted herein were based on a small number of studies only with mainly small sample sizes. This entails the risk that there may be no confidence that the statistical calculations regarding heterogeneity will be reliable. Further limitations are the subjective character of the perceived LBP outcome, the limited internal validity of the biomechanical outcome, and the non‐randomized and not‐blinded study designs as well. Additionally, the outcome MSI rate was not analyzed on a primary data basis but from available administrative data, so the findings may be subject to selection bias.^8^ The mechanical load on low back may be caused also by tasks other than by lifting and transferring alone to be done during a shift.^53^ This includes the push or pull^53^ of the mechanical devices themselves or the manual handling linked with the necessary fixing and removing of security belts for the patients before and after moving them by means of the mechanical lift and transferring devices.^55^ Thus, an exact attribution of LBP/MSI worsening/improvement to patient lifting and transferring tasks is not possible.

The literature review could not provide a consistent and reliable association between a certain frequency of mechanical patient handling transfers to be performed/needed during a certain time period, e.g., during a shift, and the break‐even point with regard to noticeable decreases of LBP and/or MSI. Such an association should in future studies ideally be combined in the same study intervention/exposure with the utmost elimination of any manual patient handling tasks in the intervention/exposure group in order to achieve practically usable results. By doing so the “…uncertainty regarding the proportion of LBP that is caused by manually lifting patients….”^9^ could be possibly avoided and the probability that beneficial LBP/MSI developments can be attributed to the use of mechanical devices only will increase. Based on a Markov analysis, Burdorf et al. predicted—based on an assumed full elimination of manual patient handling tasks—“a relative reduction of approximately 33% in the prevalence of LBP and MSD injury claims”.^9^ By doing so, in future studies this could particularly give an important practical indication for the health facilities how many mechanical patient handling devices should be used and supplied in order to reach the beneficial results for its health personnel.

## 5 CONCLUSIONS

This systematic review and meta‐analysis provide limited evidence of the impact of mechanical patient lifting and transferring devices on the reviewed outcomes. Future studies on this subject should consider a larger number of studies and should be based on larger sample sizes, granting a stronger confidence in the reliability of the statistical calculations. Future studies should rather focus on controlled observational and quasi‐experimental studies of high quality, considering the utmost elimination of any manual patient handling tasks in the intervention/exposure group and on appropriate longer follow‐ups in order to gain reliable results of impacts of the intervention on clinical LBP outcomes. These qualitative improvements of studies would facilitate and guide the future economic evaluation of capital‐intensive investments by health facilities into appropriate mechanical patient handling devices too.

## AUTHOR CONTRIBUTIONS

Conceptualization: Hans‐Udo Richarz and Timo Siepmann; Methodology: Hans‐Udo Richarz; Software: Hans‐Udo Richarz; Validation: Hans‐Udo Richarz, Jan Rahmig, and Jessica Barlinn; Formal analysis: Hans‐Udo Richarz; Investigation: Hans‐Udo Richarz, Jan Rahmig; Resources: Hans‐Udo Richarz; Data curation: Hans‐Udo Richarz; Writing ‐ original draft preparation: Hans‐Udo Richarz; Writing ‐ review and editing: Timo Siepmann, Arturo Tamayo, and Jessica Barlinn; Visualization: Hans‐Udo Richarz; Supervision: Arturo Tamayo and Jessica Barlinn; Project administration: Jessica Barlinn.

## CONFLICT OF INTEREST STATEMENT

The authors declare no conflict of interest for this article.

## DISCLOSURE

*Approval of the research protocol*: N/A. *Informed Consent*: N/A. *Registry and Registration No. of the study*: PROSPERO International Prospective Register of Systematic Reviews (N° of registration: CRD42021297165. *Animal Studies*: N/A.

## Supporting information


Appendix A.

Appendix B.

Appendix C.

Appendix D.

Appendix E.

Appendix F.

Appendix G.

Appendix H.

## Data Availability

Data are contained within the main text of the submitted research and in its supplementary materials as listed herein.
