# Supplementary material for: The impact of mechanical devices for lifting and transferring of patients on low back pain and musculoskeletal injuries in health care personnel—A systematic review and meta‐analysis
Source: J Occup Health. 2023 Sep 15;65(1):e12423. doi: 10.1002/1348-9585.12423 (PMC10502824; doi:10.1002/1348-9585.12423)
Supplement: Supplementary file 2 — Appendix B. [file JOH2-65-e12423-s001.docx]

| **#1** | "Nurses"[Mesh] OR "Health Personnel"[Mesh] OR nurse[tw] OR „nursing staff“[tw] OR „clinical nurse“[tw] OR „personnel, nursing“[tw] OR „nursing personnel“[tw]  OR „registered nurses“[tw] OR „nurse, registered“[tw] OR „nurses,  registered“[tw] OR „registered nurse“[tw] OR „health care worker*“[tw] OR „health care professional*“[tw] |
| --- | --- |
| **#2** | "Low Back Pain"[Mesh] OR "Back Pain"[Mesh] OR „Musculoskeletal Pain“[Mesh] „Back Pain, Low“[tw] OR „Low Back Pains“ [tw] OR „Pain, Low Back“[tw] OR „Pains, Low Back“[tw] OR „Lumbago“[tw] OR „Lower Back Pain“[tw] OR „Back Pain, Lower“[tw] OR „Lower Back Pains“[tw] OR „Pain, Lower Back“[tw] OR „Pains, Lower Back“[tw] OR „Low Back Pain, Postural“[tw] OR „Postural Low Back Pain“[tw] OR „Low Back Pain, Recurrent“[tw] OR „Recurrent Low Back Pain“[tw] OR „Low Back Pain, Mechanical“[tw] OR „Mechanical Low Back Pain“[tw] OR „Musculoskeletal injur*“[tw] |
| **#3** | "Self-Help Devices"[Mesh*]* OR "Moving and Lifting Patients"[Mesh] OR „Self Help Devices“[tw] OR „Self-Help Device“[tw] OR „Assistive Technology“[tw] OR „Assistive Technologies“[tw] OR „Technologies, Assistive“[tw] OR „Technology, Assistive“[tw] OR „Assistive Devices“[tw] OR „Assistive Device“[tw] OR „Devices, Assistive“[tw] OR „lifting apparatus“[tw] OR „patient lifting“[tw] OR „lifting patient*“[tw] OR „patient lift*“[tw] OR „patient repositioning“[tw] OR „moving equipment“[tw] OR „Protective Devices“[Mesh] OR „Manual Patient Handling“[tw] OR „Safe Patient Handling program*“[tw] OR „Multi-Component Intervention*“[tw] |
| **#4** | **Search (#1 AND #2 AND #3)** |

**Appendix B.** Search Strategy via PubMed (example) – Date of Search: 04.09.2021
