# Supplementary material for: The impact of mechanical devices for lifting and transferring of patients on low back pain and musculoskeletal injuries in health care personnel—A systematic review and meta‐analysis
Source: J Occup Health. 2023 Sep 15;65(1):e12423. doi: 10.1002/1348-9585.12423 (PMC10502824; doi:10.1002/1348-9585.12423)
Supplement: Supplementary file 3 — Appendix C. [file JOH2-65-e12423-s003.docx]

| **N** | **Author** | **Year** | **Title** | **Reason for exclusion** |
| --- | --- | --- | --- | --- |
|  |  |  |  |  |
| 1 | Alamgir et al. | 2009 | *Evaluation of Ceiling Lifts in Health Care Settings* | Uncontrolled Pre/Post; other target of research |
| 2 | Alamgir et al. | 2011 | *Peer Coaching and Mentoring: A New Model of Educational Intervention for Safe Patient Handling in Health Care* | Other target (peer coaching & mentoring) |
| 3 | Andersen et al. | 2014 | *Patient transfers and assistive devices: prospective cohort study on the risk for occupational back injury among healthcare workers* | Uncontrolled observational study; no clear differentiation of types of „assistive devices“ |
| 4 | Andersen et al. | 2019 | *Physical and Psychosocial Work Environmental Risk Factors for Back Injury among Healthcare Workers: Prospective Cohort Study* | Uncontrolled observational study; no relation between LBP outcome and mechanical devices as predictor variable |
| 5 | Baldessaroni et al. | 2005 | *Studio longitudinale per la valutazione dell` efficacia di misure preventive in una popolazione di operatori sanitari esposta al rischio di movimentazione manuale di pazienti* | Unclear allocation of the term „ausili“ to mechanical devices (or not) because also small aids (belts) were examined |
| 6 | Beyan et al. | 2020 | *The Effects of Multifaceted Ergonomic Interventions on Musculoskeletal Complaints in Intensive Care Units* | Multifaceted intervention: not possible to separately assign the effects to mechaniocal devices |
| 7 | Berge L. (Diss) | 2017 | *The Effect of a Multifaceted Intervention upon Staff Nurses`s Knowledge Related to Adherence to a No-lift Policy* | Different focus of the multifaceted intervention (educational intervention upon nurses knowledge of adherence to a no-lift policy |
| 8 | Budarick et al. | 2020 | *Can the Use of Turn-Assist Surfaces Reduce the Physical Burden on Caregivers When Performing Patient Turning* | Turn assist is a non-mechanical device |
| 9 | Burdorf et al. | 2013 | *Assessment of the impact of lifting device use on low back pain and musculoskeletal injury claims among nurses* | Markov simulation model using studies conducted |
| 10 | Charney et al. | 1997 | *Zero Lift Programs in Small Rural Hospitals in Washington State - Reducing Back Injuries Among Health Care Workers* | Uncontrolled Pre/Post-Design; multicomponent program (including small aids as non-friction transfer sheets); not possible to attribute effects to mechanical devices |
| 11 | Caspi et al. | 2013 | *Results of a Pilot Intervention to Improve Health and Safety for Health Care Workers* | Multicomponent Intervention; no clear/separated effect of use of lifting device on LBP outcome shown |
| 12 | Dawson et al. | 2010 | *Effect of the Workforce Initiatives Safe Handling Minimal Lift Program on Patient Care Provider Injuries, Attributable Costs and Satisfaction* | Uncontrolled Pre/Post-Design; multicomponent(WISH) program (including assistive small aids): not possible to separately assign the effects to mech. devices |
| 13 | Dennerlein et al. | 2012 | *Ergonomic Practices Within Patient Care Units are Associated with Musculoskeletal Pain and Limitations* | Multifaceted intervention: not possible to separately assign the effects to mech. devices |
| 14 | Dennerlein et al. | 2017 | *Lifting and exertion injuries decrease after implementation of an integrated hospital-wide safe patient handling and mobilisation programme* | Multifaceted intervention: not possible to separately assign the effects to mech. devices |
| 15 | Black et al. | 2011 | *Effect of Transfer, Lifting, and Repositioning (TLR) Injury Prevention Program on Musculoskeletal Injury Among Direct Care Workers* | Multicomponent Program; No clear attribution of the results to mechanical devices possible |
| 16 | D`Arcy et al. | 2012 | *Do Assistive Devices, Training, and Workload Affect Injury Incidence? Prevention Efforts by Nursing Homes and Back Injuries among Nursing Assistants* | Uncontrolled quasi-experimental study using cross-sectional data (NNAS-study); unclear whether „type of lifting“ indicated in the data used do include mechanical lifting |
| 17 | Dutta et al. | 2011 | *The effects of caregiver experience on low back loads during floor and overhead lift maneuvering activities* | Target is a comparison of floor- and ceiling lifts only |
| 18 | Dutta et al. | 2012 | *A biomechanical assessment of floor and overhead lifts using one or two caregivers for patient transfers* | Target is a comparison of floor- and ceiling lifts only |
| 19 | Dutta T. (Diss.) | 2012 | *Preventing Back Injury in Caregivers* | Target is a comparison of floor- and ceiling lifts only |
| 20 | Edlich et al. | 2004 | *Prevention of disabling back injuries in nurses by the use of mechanical patient lift systems* | No research study |
| 21 | Engkvist et al. | 2006 | *Evaluation of an intervention comprising a No Lifting Policy in Australian hospitals* | Mulicomponent study including small aids; not possible to separately assign the effects to mech. devices |
| 22 | Fragala et al. | 2014 | Improving the Safety of Patient Turning and Repositioning Tasks for Caregivers | Comparison between a „Turning & Repositioning System“ (TAP) and a small aid (draw sheet) |
| 23 | Fujishiro et al. | 2005 | *The Effect of Ergonomic Interventions in Healthcare Facilities on Musculoskeletal Disorders* | Uncontrolled Pre/Post-Design |
| 24 | Garg et al. | 1991 | *A biomechanical and ergonomic evaluation of patient transferring tasks: bed to wheelchair and wheelchair to bed* | No impact measurement by comparing mechanical hoists and manual lifting to compressive/shear spinal load |
| 25 | Garg et al. | 1992a | *An ergonomic evaluation of nursing assistants` job in a nursing home* | No identifiable comparison between (chair) lift and manual handling on on perceived LBP excertion |
| 26 | Garg et al. | 1992b | *Reducing back stress to nursing personnel: an ergonomic intervention in a nursing home* | No impact measurement by comparing mechanical hoists and manual lifting to compressive/shear spinal load |
| 27 | Garg et al. | 1994 | *Prevention of back injuries in healthcare workers* | Uncontrolled Pre/post-design (Field Study) and no biomechanical analysis regarding „hoists“ (biomechanical study) |
| 28 | Garg et al. | 2012 | *Long-term Efficacy of an Ergonomics Program That Includes Patient-Handling Devices On Reducing Musculoskeletal Injuries to Nursing Personnel* | Uncontrolled Pre/Post-Design |
| 29 | Guthrie et al. | 2004 | *A patient lifting intervention for preventing the work-related injuries of nurses* | Intervention period not clearly identifiable being important for ITS-studies; unclear whether results are attributed to „lift teams“ or „new mechanical lifting equipment“ |
| 30 | Gilchrist et al. | 2020 | *Prevalence of musculoskeletal low back pain among registered nurses: Results of an online survey* | Uncontrolled cross-sectional study |
| 31 | Gold et al. | 2017 | *Predictors of low back pain in nursing home workers after implementation of a safe resident handling programme* | Uncontrolled observational study; Multifaceted intervention: not possible to attribute the effects to mechanical devices |
| 32 | Hartvigsen et al. | 2005 | *Intensive education combined with low tech ergonomic intervention does not prevent low back pain in nurses* | Examination limited to „low tech ergonomic aids“ as, e.g. plastic sheets, slings, only |
| 33 | Hinton et al. | 2009 | *Patient Lift Systems May Not Prevent Injury Claims in Rehabilitation Nurses and Therapists* | Uncontrolled Pre/Post-Design |
| 34 | Hodder et al. | 2009 | *Continuous assessments of work activities and posture in long-term care nurses* | Uncontrolled observational study; outcome „trunc posture“ only does not fit |
| 35 | Hodgson et al. | 2013 | *Patient Handling in the Veterans Health Administration – Facilitating Change in the Health Care Industry* | Uncontrolled decriptive study; no usable relation between the use of mechanical devices and LBP/MSI detectable |
| 36 | Huffmann et al. | 2014 | On SHiPs and Safety: A Journey of Safe Patient Handling in Pediatrics | Uncontrolled Pre/Post-design; multicomponent (SHP) program: not possible to assign the effects to mech. devices |
| 37 | Holtermann et al. | 2015 | *Does rare use of assistive devices during patient handling increase the risk of low back pain? A prospective cohort study among female healthcare workers* | Uncontrolled cohort study; unclear definition of „assistive devices“, therefore not possible to attributethe effects to mech. devices |
| 38 | Knibbe et al. | 2012 | *Flying through the hospital: efficiency and safety of an ergonomic solution* | Uncontrolled Pre/Post-design; effects of stretcher slings on effectivity/security are examined only |
| 39 | Kutash et al. | 2009 | *The Lift Team`s Importance to a Successful Safe Patient Handling Program* | Benefits of „lift teams“ examined only instead of the availability of a large patient transfer/lift (mechanical) equipment |
| 40 | Lavender et al. | 2016 | *Biomechanical loads on the spine as patients are prepared for mechanical transfers: have patient lifts completely solved the problem?* | Abstract of an conference paper) only |
| 41 | Li et al. | 2004 | *Use of mechanical patient lifts decreased musculoskeletal symptoms and injuries among heaalth care workers* | Uncontrolled Pre/Post-design |
| 42 | Lee et al. | 2013 | *Musculoskeletal pain among critical-care nurses by availability and use of patient lifting equipment: An analysis of cross-sectional survey data* | Uncontrolled cross-sectional study |
| 43 | Lee et al. | 2010 | *Factors Associated With Safe Patient Handling Among Critical Care Nurses* | Uncontrolled cross-sectional study; not possible to attribute the effects to mechanical devices („lift devices or lift teams“/see Table IV) |
| 44 | Lee et al. | 2019 | *Impact of California`s safe patient handling legislation on musculoskeletal injury prevention among nurses* | Uncontrolled cross-sectional surveys (2013/2016); multicomponent (SPH) program: not possible to attribute the effects to mechanical devices |
| 45 | Lee et al. | 2020 |  | Data from uncontrolled cross-sectional study; Comparison between ceiling- and floor lifts only |
| 46 | Lee et al. | 2015 | *Musculoskeletal Symptoms in Nurses in the Early Implementation Phase of California`s Safe Patient Handling Legislation* | Uncontrolled cross-sectional study |
| 47 | Lee et al. | 2017 | Safe patient handling and lift use among hospital nurses: A cross-sectional study | Uncontrolled cross-sectional study |
| 48 | Lipscomb et al. | 2012 | *Evaluation of direct workers`compensation costs for musculoskeletal injuries surrounding interventions to reduce patient lifting* | Uncontrolled (analytical) quasi-experimental study; no relation to LBP; only cost comparison between patient handling and non-patient handling activities |
| 49 | Marras et al. | 2009 | *Lumbar spine forces during manoeuvring of ceiling-based and floor-based patient transfer devices* | Population not appropriate (university students and not nurses/HCWs) |
| 50 | Miura et al. | 2021 | *Successful Use of the Hybrid Assistive Limb for Care Support to Reduce Lumbar Load in a Simulated Patient Transfer* | Uncontrolled quasi-experimental study; population not appropriate (volunteers and not nurses/HCWs) |
| 51 | Nelson et al. | 2006 | *Development and evaluation of a multifaceted ergonomics program to prevent injuries associated with patient handling tasks* | Uncontrolled Pre/Post-design; no relation between lift equipment as part of the multifaceted program and LBP/MSI |
| 52 | Owen et al. | 1999 | *Reducing Perceived Physical Stress While Transferring Residents* | Different interventions; no separate attribution of effects to mechanical lift-part possible |
| 53 | Ovayoglu et al. | 2014 | Frequency and severity of low back pain in nurses working in intensive care units and influential factors | Uncontrolled cross-sectional study; unclear meaning of „any aiding equipment“ |
| 54 | Park et al. | 2009 | *Impact of Publicly Sponsored Interventions on Musculoskeletal Injury Claims in Nursing Homes* | Unclear meaning of „equipment purchase“ |
| 55 | Powell-Cope et al. | 2014 | *Effects of a National Handling Program on Nursing Injury Incidence Rates* | Uncontrolled Pre/Post-design; with regard to the term „ceiling lifts & other new technologies“ unclear whether non-mechanical technologies are included or not |
| 56 | Lim et al. | 2011 | *Evaluating repeated patient handling injuries following the implementation of a multi-factor ergonomic intervention program among health care workers* | Multi-factor program: not possible to attribute the effects to mechanical devices |
| 57 | Lloyd et al. | 2006 | *Friction-Reducing Devices for Lateral Patient Transfers* | No study on nurses/HCWs; examination of friction-reducing lateral transfer technologies/techniques only |
| 58 | McGill et al. | 2005 | *Transfer of the horizontal patient: The effect of a friction reducing assistive device on low back mechanics* | Uncontrolled quasi-experimental study; No examination of mechanical devices but of 3 - non-mechanical - friction reducing devices only |
| 59 | Mitchell et al. (Diss.) | 2008 | *Towards the identification of modifiable personal predictors of low back pain in nursing students* | No clinical study examining the relation between mechanical devices and LBP/MSI |
| 60 | Rasheed et al. | 2017 | *Frequency of low back pain among nurses working in Jinnah hospital Lahore* | Uncontrolled cross-sectional study examining the frequency of LBP in nurses only |
| 61 | Risor et al. | 2017 | *A multi-component patient-handling intervention improves attitudes and behaviors for safe patient handling and reduces aggression experienced by nursing staff: A controlled before-after study* | Multi-component intervention: not possible to attribute the effects to „patient-handling equipment“ which was in addition not differentiable, too |
| 62 | Roth et al. | 1993 | *Evaluation of a Unique Mechanical Client Lift* | Comparison belt lift/manual handlings not clearly related to LBP or MSI |
| 63 | Schoenfisch et al. | 2013 | *Musculoskeletal injuries among hospital patient care staff before and after implementation of patient lift and transfer equipment* | Uncontrolled cohort study |
| 64 | Skela-Savic et al. | 2020 | *Nurses`work characteristics and self-assessment of the work environment - Explorative cross-sectional study* | Uncontrolled cross-sectional study; unclear relatedness between application of hoists and LBP |
| 65 | Smedley et al. | 1995 | *Manual handling activities and risk of low back pain in nurses* | Uncontrolled cross-sectional study |
| 66 | Smedley et al. | 1997 | *Prospective cohort study of predictors of incident low back pain in nurses* | Uncontrolled cohort study |
| 67 | Smedley et al. | 2003 | *Impact of ergonomic intervention on back pain among nurses* | Multi-factor intervention; unclear evaluation of the relatedness between hoists and prevalence/change in prevalence as outcome |
| 68 | Smedley et al. | 2005 | *Epidemiological Differences Between Back Pain of Sudden and Gradual Onset* | Explorative analysis from data from 2 longitudinal studies; comparison of „baseline“-values of intervention with/without „hoist or other lifting device“; unclear meaning of „other lifting device“ |
| 69 | Stevens et al. | 2019 | Mechanisms for reducing low back pain: a mediation analysis of a multi-faceted intervention in workers in elderly care | Unclear meaning of „assistive devices“, examination of an possible mediator-nature of the intervention in relation to LBP only |
| 70 | Stevens et al. | 2013 | *Creating a culture of safety for safe patient handling* | Abstract available only; volume 2013 of the journal not avilable |
| 71 | Samaei et al. | 2017 | *Effects of patient-handling and individual factors on the prevalence of low back pain among nursing personnel* | Uncontrolled cross-sectional study; no relatedness between mechanical hoist/non-mechanical lifting and LBP/MSI identifiable |
| 72 | Trinkoff et al. | 2003 | *Workplace Prevention and Musculoskeletal Injuries in Nurses* | Uncontrolled cross-sectional study |
| 73 | Vinstrup et al. | 2020b | *Physical exposure during patient transfer and risk of back injury & low-back pain: prospective cohort study* | Uncontrolled cohort study |
| 74 | Weiner et al. | 2017 | *Repositioning a passive patient in bed: Choosing an ergonomically advantageous assistive device* | Comparison of the impact of three non-mechanical decives on repositioning tasks only |
| 75 | Wiggermann et al. | 2021a | *Effect of Repositioning Aids and Patient Weight on Biomechanical Stresses When Repositioning Patients in Bed* | Comparison of the impact of three non-mechanical decives on repositioning tasks only |
| 76 | Noble et al. | 2018 | *Barriers to the Use of Assistive Devices in Patient Handling* | Examination of use/frequency of „assistive devices“ only |
| 77 | Zadvinskis et al. | 2010 | *Effects of a Multifaceted Minimal-Lift Environment for Nursing Staff: Pilot Results* | Multi-faceted intervention: not possible to attribute the effects to the unclear nature of the intervention „Engineering controls (minimal- lift equipment)“ |
| 78 | Zhou et al. | 2021 | *The effect of hospital bed features on physical stresses on caregivers when repositioning patients in bed* | Compared 3 „assistive bed features“ are non-mechanical devices |
| 79 | Santaguida et al. | 2005 | *Comparison of cumulative low back loads of caregivers when transferring patients using overhead and floor mechanical lifting devices* | Comparison between 2 types of mechanical lifts on L5/S1 compressive load only |
| 80 | Silverwood et al. | 2006 | *Reduction of musculoskeletal injuries in intensive care nurses using ceiling-mounted patient lifts* | Publishing journal „Dynamics“ not available |
| 81 | Wiggermann et al. | 2021b | *The effects of hospital bed features on physical stresses on caregivers when repositioning patients in bed* | Comparison between turn-assist and hand force on repositioning tasks only |
| 82 | Bukhari et al. | 2019 | *Low Back Pain Among Nurses Working In Different Hospitals* | Uncontrolled cross-sectional study; no mechanical devices examined |
| 83 | Waters et al. | 2012 | *Ergonomic Assessment of Floor-based and Overhead Lifts* | Biomechanical Study on a comparison of floor- and ceiling lifts only |
| 84 | Schoenfisch et al. | 2019 | *Use of Assistive Devices to Lift, Transfer, and Reposition Hospital Patients* | Target of the study limited to the use of lift/transfer devices only |
| 85 | Restrepo et al. | 2018 | *Safe Lifting Programs at Long-Term Care Facilities and Their Impact on Workers` Compensation Costs* | Other target/Research Question |
| 86 | Coenen et al. | 2013 | *Cumulative mechanical low-back load at work is a determinant of low-back pain* | Examination of manual handling only |
| 87 | Muto et al. | 2008 | *Effect of Nursing Assistance Tools on Preventing Musculoskeletal Pain among Staff in Schools for Diasabled Children* | Examination of small aids only |
| 88 | Warming et al. | 2009 | *Little effect of transfer technique instruction and physical fitness training in reducing low back pain among nurses: a cluster randomised intervention study* | Examination of pure manual TTPT-program (manual transfer technique/physical fitness training) vs usual (manual) routine (control group) |
| 89 | Evanoff et al. | 2003 | *Reduction in Injury Rates in Nursing Personnel Through INtroduction of Mechanical Lifts in the Workplace* | Non-eligible study design |
