# Supplementary material for: The impact of mechanical devices for lifting and transferring of patients on low back pain and musculoskeletal injuries in health care personnel—A systematic review and meta‐analysis
Source: J Occup Health. 2023 Sep 15;65(1):e12423. doi: 10.1002/1348-9585.12423 (PMC10502824; doi:10.1002/1348-9585.12423)
Supplement: Supplementary file 4 — Appendix D. [file JOH2-65-e12423-s005.zip › Appendix D_Table A1_Main characteristics of RCT & Quasi-experimental studies included_26.03.2023.docx]

| **Table A1**. Main characteristics of RCT- & Quasi-experimental studies included | | | | | | |
| --- | --- | --- | --- | --- | --- | --- |
| **Study (Year**  **of Publication)/**  **[Quality Score]** EPOC/RoB 2.0 Cochrane  [2] = Low Risk/  [0] = High Risk] | **Design** | **Sample** | **Population** | **Intervention/Control** | **Outcome (Impact)** | **Results** |
| **Alamgir** (2008) **[2]** | (Single) Interrupted Time Series Study (uncontrolled) | N=455  (586 MSI claims) | 3 long-term care facilities (A-C) in Vancouver/Canada | Pre-/Post Implementation of ceiling lifts | Reduction of MSI rate/frequency; working days lost/compensation claims; Cost-Benefit-Analysis | -Frequency MSI Pre/Post 422/164  -MSI-rate (bed) Pre/Post 0.16/0.09 -Relative Risk MSI (RR); 95% CI:  Pre (1996-2001/Post(2003-2005)-Phases:  Pre 1.00 Post 0.56 (0.47-0.67) P=<0.0001  - Trend estimate over total period (1996-2005):  -0.0983 (-0.1270 until -0.0696)/95% CI P=<0.000 |
| **Anyan** (2013) **[2]** | (Single) Interrupted time Series Study (uncontrolled) | N= 45  (cases=injured staff members during study period) | Female/male staff menbers in a burn trauma intensive care unit in USA | Pre-/Post Installation of 5 overhead lifting systems (OLS) in 12 ICU rooms | Reduction of (Lower) Back Injury rate; Incidence rate | Frequency (Lower) Back Injury  Pre/Interim/Post 33/11/1  MSI Incidence Rate (per 100 employee years):  Pre/Interim 6.5/5.8; p=0.775  Pre/Post 6.5/0.3; p= < 0.001 |
| **Collins**  (2004) **[2]** | (Single) Interrupted Time Series (uncontrolled) | N=1728 | Nurses from 6 nursing homes (USA) covering a total of 552 beds | Intervention (MSI prevention program): Implementation of mechanical lifts (full-body- and stand-up lifts), friction reducing sheets for repositioning in bed, each handling category including initial and continued training on lift equipment & zero lift policy. | Reduction in MSI-injury rates; workers compensation costs, lost work day injury rates; restricted work day rates; resident assaults on caregivers (p.a. each); Cost Benefit Analysis | Use of mechanical lifting equipment for Task „Lifting resident off the floor“ Pre/Post:  MSI (Frequency) Rate /per 100 FTE):  Pre: 0.54 (5 obs./129)  Post: 0.32 (3 obs./56)/41% reduction  Task „In/out of bed-bed to chair & chair to bed  Pre: 3.80 (35 obs)/129  Post: 1.49 (14 obs/56_61% reduction |
| **Chhokar**  (2005) **[0]** | (Single) Interrupted Time Series Study  (uncontrolled) | N=65 (total MSI claims)  N=30 (lifting & transferring MSI claims)  N=35 (repositioning MSI claims) | HCWs (USA) of an extended care facility covering a total of 125 beds | Intervention: Implementation of mechanical lifts (65 ceiling lifts for 125 beds/3 bathtubs) training of use of the lift equipment & no unsafe manual lift policy.  Control: no control | Reduction in MSI-injury claims; workers compensation costs, days lost, direct MSI cost (Cost Benefit Analysis) | Total No. of MSI claims („lifting & transferring“):  Pre/Post 30/10  Frequency of MSI claims: („lifting & transferring“):  Correlation r²:  Pre/Post 0.463/0.729  Slope (b)  Pre/Post 1.14/-0.89  t-score 3.021  p-value (95% CI) 0.006 |
| **Engst**  (2005) **[2]** | Interrupted Time Series Study (controlled | N= 50 (in total)  Intervention N=34  Control N=16 | HCWs of extended care facilities (IG: 75-bed extended care unit; CG: similar 75-bed extended care unit) from the same hospital in Vancouver, Canada | Intervention:  -Implementation of a mechanical ceiling lift pr program by OHSAH  - During intervention period (6 months) staff training on use of new ceiling lifts (1-hour training session)  - introduction of a „no-unsafe manual lift policy“  Control:  No implementation of the intervention, i.e. ceiling lifts. Only 3 mechanical floor lifts and one sit-stander available | -Total No. (frequency) of MSI for „lifting & transferring“ as well as for repositioning patients job satisfaction, preferred resident handling methods,  -perceived staff perception on risk of LBP injury on the basis of (scale-based) questionnaires  -CBA on ceiling lift program | Frequency of MSI  -(lifting & transferring)  Intervention/Control  Pre: 5/5 Post: 5/5  -(repositioning)  Intervention/Control  Pre: 7/4 Post: 5/5  Risk of MSI injury  on lower back p<0.001 (no effect size indicated) |
| **Fragala**  (2012) **[0]** | Pilot Study  with a Pre-Post-Design (controlled) | N= N/A (with regard to staff at risk) | Caregivers of 2 units of the Mayflower LTC facility in Plymouth, MA/USA | Intervention:  Installation of CASE-program consisting of mechanical equipment (4 full sling lifts & 1 stand-assist lift), education & training on use of lifts with vendor demonstration; Pre-/Post evaluation (by questionnaires).  Control: no CASE-program provided | Reduction of Musculoskeletal Injuries (MSI) in total No. (frequency) | MSI No (frequency)  Intervention Group:  Pre: 4  Post: 0  Control Group:  Pre: 2  Post: 3 |
| **Knibbe**  (1999) **[0]** | Quasi-experimental Pre-Post Design  (controlled) | N=378  IG: N=139  CG: N=239 | Home Care Nurses working at Rotterdam/Netherlands dealing with manual transfers of patients | IG:Implementation of 40 mechanical hoist lifts during 1 year study period; additional provision of training; 12 trained `lifting coordinators & use of ergonomic assessment forms  CG: continuation of „normal routines“ with 2 hoists already available | 12-month prevalence of back pain assessed by a pre/post survey for 7 consecutive days respectively | 12-month prevalence back pain  **Intervention:**  Pre 74% (N=132)/Post 64% (N=N/A)  p < 0.05 (McNemar)  **Control:**  Pre 62% (N=223)/Post 66% (N=N/A)  P < 0.05 (Chi-square) |
| **Miller**  (2006) **[0]** | Quasi-experimental Pre-/Post Design  (controlled) | N=74 (total)  IG N=45  CG N=29 | Frontline care staff (registered,-licensed nurses+Care Aides from 2-longterm care facilities (IG: 63 beds facility; CG:100 beds facility) at Vancouver/Canada | Intervention:  (Beside already 4, in the Pre-phase available, mechanical floor lifts) implementation of 6 portable ceiling lifts+4 further ceiling lift motors lifts (after 8 months) including one-time (1 h) training session by the lift manufacturer on the use of the lifts.  Control:  No ceiling lifts: only 4 mechanical floor lifts in operation (as in the IG) during the study period | Self-reported MSI symptom survey (including lower back) assessed by 5 point scale, MSI frequency, adjusted lost day No of injuries/injury rates workers`compensation costs | **No of MS injuries Pre/Post**  Intervention:  Pre 1 (1st year intervention): 3  Pre 2 (2nd year intervention): 2  Post (3rd year): 1  Control:  Pre 1: 8  Pre 2: 8  Post: 14  **Perceived risk of LBP**  Control: (no ceiling lift):  CG 1: Manually (1 person):  Mean* (SD): 7.18 (2.53)  F=32.09/p=0.0001 (0.05 level)  CG 2: Manually (2 persons):  Mean* (SD): 5.94 (2.79)  F=11.15/p=0.004 (0.05 level)  Intervention (ceiling lift):  Compared with CG 1:  Mean* (SD): 3.29 (2.49)  Compared with CG 2:  Mean* (SD): 3.50 (2.42)  *mean=average score from the responses based on a 1-10 scale |
| **Owen**  (2002) **[2]** | Quasi-experimental Pre-Post Design (controlled); 2 studies combined: Study I Pre-/Post Invervention Study and Study II: Post-5 years follow up study | N=57 (total)  IG N=37  CG N=20 | Nurses from 2 US-hospitals working in medical-surgical units, carrying out patient handling tasks | Intervention:  Implementation of 5 assistive devices based on a preceding laboratory study consisting of 2 types of mechanical lifts for transferring patients from bed to chair and vice versa and walking belts and friction-reducing sheets for other tasks  Control: only the „usual methods of lifting and transferring of patients“ were used | **Study I** (Pre-/Post; 18 months each)  Perceived exertion (including lower back) felt by nursing staff assessed by 5 point Likert-type scale (Borg scale, 1982), lower back injuries, lost work days and restricted/light workdays  **Study II** (Post 5 years follow up):  Back Injuries, lost workdays and restricted/light work days | **Study I:**  -Rating of perceived exertion** on LBP:  Mechanical lift types 1 and 2 (1st task: transfer bed to wheelchair/commode and 2n task: Wheelchair/commode to bed):  1st task:  Intervention: mean (SD)=0.5 (0.8)*  Control: mean (SD)=3.5 (1.2)  2n task:  Intervention: mean (SD)=0.7 (0.9)*  Control: mean (SD)=3.5 (1.3)  *significant at p<0.001  **Rating of perceived exertion (0=no exertion; 10= extremly heavy, maximal excertion  -Back injuries 18 months Pre-Phase: 20 (Data Intervention/Control Group were comparable)  -Back Injuries 18 months Post-Phase: 12  (Data N.N. of control group)  **Study II**:  Back Injuries (year 1= 1/year 2= 3/year 3= 16 (61%)/year 4= 3/year 5= 3 |
| **Ronald**  (2002) **[2]** | Interrupted Time Series Design  (uncontrolled) | N=129 | 73 long term care aides, 30 registered nurses and 5 activity aides in an extended care hospital unit in British Columbia/Canada | Implementing 65 new ceiling lifts in addition to the existuing equipment during implementation phase between April and August 1998. In addition trainings in the lift use, musculoskeletal injury prevention program courses in June 1999 to RNs and between September and November 1999 ffor LTCA, no manual lift policy in March 1998 plus a new transfer belt policy | MSI rates collected retrospectively from April 1995 to March 1998 preceding the installation of the ceiling lifts and from August 1998 to March 2000 post-installation of ceiling lifts.  MSIs were calculated for two Pre-Phases (April 1 to September 19, 1996 and September 20,1996 to March 31,1998) and the Post-Phase (August 21,1998 to March 31, 2000) | MSI rate* for tasks (lifting/transferring) being completed ad time of MSI:  -Lifting or transferring overall:  Pre 1: 14.5/Pre 2:17.9/Post 8.1  -Lifting task  Pre-Phase 1/Pre-Phase 2/Post Phase:  77.0/8.2/4.3  -Transferring Task:  Pre-Phase 1/Pre-Phase 2/Post-Phase  7.5/9.7/3.8  Overall MSI rate for post-lifting/transferring patients:  < 58 % (p=0.011)  * per 100.000 worked hours |
| **Spiegel**  (2005) **[2]** | Interrupted Time Series Design (uncontrolled) | N=61 (total MSI claims  N=24 (lifting & transferring MSI | HCWs from an extended care unit of a hospital in British Columbia/Canada | Implementation of a „Resident Lifting System Project“ consisting of an installment of 65 ceiling lifts, provision of trainingfor use and introduction of a „no manual lift policy“ | MSI injuries (No/%); Cost-Benefit-Analysis (CBA) MSI rates collected retrospectively from April 1, 1997 to March 31,1998 (Pre-Phase) preceding the installation of the ceiling lifts (April 1, 1998 to August 20, 1998) and from August 21, 1998 to August 20, 1999 (Post-phase) | MS injuries from „lifting & transferring“*:  Pre/Post: 24/10 (42% decrease)  p=0.011  * MSIs were calculated based on the No of MSIs per 100.000 |
| **Yassi**  (2001) **[0]** | 3-arm-randomized controlled study | N= 346 (in total)  Arm A: 103/followed 82 (=80%)  Arm B: 116/followed 85 (=73%)  Arm C: 127/followed 94 (=74%) | Nurses & Unit Assistants from 9 hospital wards(3 surgical, medical & rehabilitation each) at Health Sciences Center (HSC) in Manitoba/Canada | **I 1:** Intervention Group (Arm B)  “Safe Lifting Program“: 1 mechanical total body lift, transfer belts in each room, 2 large/4 small sliding devices; 3-hrs education on back care, patient assessment, handling techniques and on use of equipment available  **I 2**: Intervention Group 2 (Arm C)  „No-strenous lifting program“ each arm provided with new mechanicel patient lift & transfer equipment (mechanical total body lifts; sit-stand lifts plus set of sliding devices for each room); 3-hrs education as Group I 1  **C:** Control Group (Arm A)  „Usual Practice Program: as per HSC practice: available 1 mechanical total body lift on a ward and sliding devices, each only on request | Outcomes (with regard to LBP and MSI):  **(1)** Work-related LBP self-ratings (past week) over time (baseline/6 months/12 months);  **(2)** Comparison of changes (6 months/12 months) in self-ratings of work-related LBP (past week);  **(3)** LBP disability (Owestry questionnaire) over time (baseline/ 6 months/12 months);  **(4)** MSI No., MSI Rate, MSI (total) cost; Cost per time loss injury. |  |
| **Yassi** (2001): **Results (1) – (4);** see Outcomes  **Arm A Arm B Arm C**  **(1)** Baseline 6 months 12 months Baseline 6 months 12 months Baseline 6 months 12 months  20.2 ± 24.2 21.7 ± 24.8 24.1 ± 26.5 35.9 ± 29.7 33.0 ± 29.4 27.1 ± 24.9 26.5 ± 28.1 21.6 ± 21.7 24.2 ± 25.4  *All pairwise p=>0.05* *6 months vs. 12 months (p=0.034) 0 vs 12 months* *All pairwise p=> 0.05*  **(2) Changes at 6 months** **Changes at 12 months**  Arm A Arm B Arm C Arm A Arm B Arm C  3.8 ± 27.5 -0.6 ± 24.1 -5.2 ± 28.8 1.0 ± 26.1 -6.5 ± 27.6 -3.3 ± 27.0  **(3)** **Arm A** **Arm B** **Arm C**  Baseline 6 months 12 months Baseline 6 months 12 months Baseline 6 months 12 months  5.4 ± 9.0 5.8 ± 9.3 6.4 ± 10.6 7.2 ± 8.8 7.2 ± 9.5 6.7 ± 8.7 5.7 ± 8.0 6.7 ± 9.0 5.4 ± 7.6  **(4) Arm A** (Study Year) **Arm B** (Study Year) **Arm C** (Study Year)  **MSI** (in total)  **MSI** (in total) **MSI** (in total)  No MSI MSI rate No MSI MSI rate No MSI MSI rate  20 7.6 13 5.3 17 6.1 | | | | | | |
