# Supplementary material for: The impact of mechanical devices for lifting and transferring of patients on low back pain and musculoskeletal injuries in health care personnel—A systematic review and meta‐analysis
Source: J Occup Health. 2023 Sep 15;65(1):e12423. doi: 10.1002/1348-9585.12423 (PMC10502824; doi:10.1002/1348-9585.12423)
Supplement: Supplementary file 4 — Appendix D. [file JOH2-65-e12423-s005.zip › Appendix D_Table A2_Main characteristics for possible Cost-Benefit Analysis-Outcomes_26.03.2023.docx]

| **Table A2.** Main characteristics for possible **Cost-Benefit-Analysis (CBA)-Outcomes** | | | | | |
| --- | --- | --- | --- | --- | --- |
| **Study (Year of Publication)** | **Study Duration**  (months) | **Days Lost (DL)** per MSI (No.)/DL-Rate/DL-RR post)  Pre/Post | **Lost work day injury**  -Rate  -RR (95%CI) | **Total MSI claim costs** (currency) | **Intervention costs/Total savings/Payback period** |
| **Alamgir** (2008) | **Pre** 1996-2001 72  **Intervention** 2002 (assumed)  **Post** 2002-2005 48 | **Frequency (No)**  Pre 14.768  Post 7315  **Relative Risk (RR);** 95% CI  Pre 1.0  Post 0.72 (0.70-0.74) P=<0.0001 | N/A | **Pre**  2.543.087*  **Post** 872.477*  *in Can$; adjusted for inflation & time value of money | Intervention cost:  1.081.410*  (110 lifts plus training & maintenance cost)  Total mean savings p.a.**  2.099.943,93  Payback Period (Method 1):  6.18 (direct savings only) or 3.09/2.06 (equal/double direct savings=indirect savings) or  Payback Period (Method 2):  6.39 (direct savings only) or  3.20/2.13 (equal/double direct savings=indirect savings)  *in Can$/**direct+indirect cost |
| **Anyan**  (2013) | Pre 64  Interim 24  Post 42 | Pre 1.148  Interim 70  Post 0 | N/A | Pre 315.054*  Interim 14.105*  Post 52* | 35.000* in total (5 OLS)  (7.000 per OLS unit)  *US$ |
| **Collins**  (2004) | Pre 1995-1997 36  Intervention in 01/1998  Post 1998-2000 36 | Days lost from MSI  Pre 488  Post 229  Days lost frequency rate (per 100 FTE)  Pre: 5.8  Post 2.0 | Lost work day injury rate/95% CI:  RR 0.34 (0.20-0.60) | (1)Total workers cost compensation:  Pre: US$ 441.670  Post: US$ 277.060  (2 )No. of claims/1000 h/rate per 100 FTE:  Pre: 129/1841.2 (14.0)  Post: 56/1873.5/(15.9)  (3)Risk Ratio (RR):  Pre: 1.00 (adjusted)  Post: 0.39adjusted; (0.29-0.55), 95% CI  (4)(Direct) total savings:  US$ 164.609,  i.e. US$ 55.000 p.a.  CBA = Return on Investment (ROI <3 years) | (Initial) Equipment cost (including training):  US$ 158.556 |
| **Chhokar**  (2005) | Pre 1995-1997 36  Intervention Period (04/1998-09/1998) 6  Post 1998-2001 36 | Days lost (MSI/6 months) for „lifting & transferring“ MSI claims:  Correlation r²: 0.640 to 0.345  Slope (b): 128.37 to -58.09  t-score: 2.249  p-value: 0.007; 95% CI | N/A | Total workers cost compensation (6 months):  Pre/Post r²=0.429-0.373/b=13326 to - 5353.5/t-score=2.213/p-value=0.026  Baseline claim cost: US$ 238.166 per year | Initial investment Intervention:  US$ 344.323  Total (annual) savings during 3-years post-intervention: US$ 412,754 relative to baseline MSI claim costs per year. Assuming an increase of MSI claim cost in 3 years-Post phase the extrapolated intervention cost=US$ 1.559.349 and totalcost saving during this period=US$ 1.257.605 with a ROI of 0.82 per year |
| **Daynard**  (2001) | Data from 36 months  (retrospective) | N/A | N/A | N/A | N/A |
| **Engst**  (2005) | 48 months (in total)  Pre: 01/1999-10/200 21  Intervention Period (11/2000-03/2001) 6  Post 04/12/2002 21 | N/A | N/A | Intervention** (-68.3%)  Pre 37.140*  Post 11776*  Control** (+68.3%)  Pre 12738*  Post 21438*  *in US$  ** „Lifting & Transferring“ | -Investment „Lifting & Transferring claims (in 2001/2002):  US$ 284.297  **-**Direct Savings  US$ 14.493  -Payback period estimate 6.5 years |
| **Fragala**  (2012) | 12 months  (for both groups) | Work Days lost from MSI (4 MSI/12 months)  Intervention: Pre/Post: 236/0  Control: Pre/Post: 0/0  Restricted work days from MSI  Intervention: Pre/Post: 2/0  Control: Pre/Post: 0/5 | N/A | N/A | N/A |
| **Knibbe**  (1999) | 12 months  (for both groups) | N/A | N/A | N/A | N/A |
| **Miller**  (2006) | 36 months (total)  Pre 1: 12 months  Pre 2: 12 months  Post: 12 months | Work Days lost from MSI (from Pre 2 to Post):  Intervention: 18 days  Control: 499 days | N/A | MSI claim costs* (from Pre 2 to Post):  Intervention: < 70%** decrease (Pre 2: ca.US$ 3.000**/Post: ca.US$ 1.000**)  Control: > 45%** increase (Pre 2: ca. US$ 28.000**/Post: ca. US$ 41.000**  * MS injury cost limited to the „Lift & Transfer“-task  **own estimation of total US$ and percentages of increase/decrease derived from Figure 3 of the study | N/A |
| **Owen**  (2002) | 86 months (total)  Study I:  Pre 1: 18 months  Pre 2: 18 months  Study II (follow-up)  Post: 60 months | Lost Work Days (LWDs)  Study I:  **Intervention:** Pre/Post= 64/3  **Control:** Pre/Post= 64  Study II (5 years follow up): in total 87 LWDS: year 1= 0/year 2= 67 (64, however, caused by one nurse)/year 3= 2/year 4= 18/year | N/A | N/A | N/A |
| **Ronald**  (2002) | 48 months (total)  Pre 1: 6 months  Pre 2: 18 months  Intervention: 5 months  Post: 19 months | N/A | N/A | N/A | N/A |
| **Spiegel**  (2002) | 24 months (Pre/Post)  Pre: 12  Intervention: 5  Post: 12 | N/A | N/A | **MSI related* claim costs:**  Pre (adj.): 86.432**  Post: 27.150**  Net Reduction Pre/Post:  59.282 (= ca. 32%)  **Payback Period Estimate***=3.85 years  **Cost Benefit Ratio**: 2.53  (with regard to a projected 12 year life span for the equipment resulting in savings of $Ca 872.372 savings)  **Return on Investment** of ca. 8.1% p.a.  *for „Lift & Transfer“ related MSI claims only  *currency in $Ca | 344.323*  *$Ca |
| **Yassi**  (2001) | 12 months | N/A | Arm A Arm B Arm C  No  7 8 4  Rate  2.7 3.2 1.5  Cost  3.426* 2.522* 3.376*  *$Ca | Arm A Arm B Arm C  23.984* 20.179* 13.502*  *$Ca | N/A |
