# Supplementary material for: The impact of mechanical devices for lifting and transferring of patients on low back pain and musculoskeletal injuries in health care personnel—A systematic review and meta‐analysis
Source: J Occup Health. 2023 Sep 15;65(1):e12423. doi: 10.1002/1348-9585.12423 (PMC10502824; doi:10.1002/1348-9585.12423)
Supplement: Supplementary file 5 — Appendix E. [file JOH2-65-e12423-s008.docx]

**Appendix E** – **Funnel Plots**

**Table A1.** Funnel Plot – **MSI rate** (calculated via Comprehensive Meta-Analasis); random, observed+imputed values

**Table A2.** Funnel Plot – **LBP perceived** (calculated via STATA 17); random, Hedge`s g, observed+imputed values


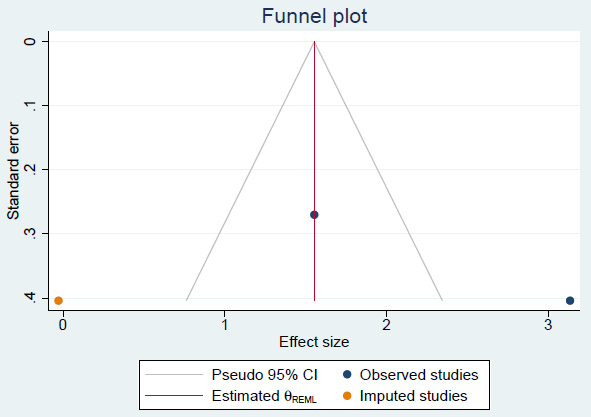


**Table A3.** Funnel Plot – **LBP peak compressive spinal load** (calculated via Comprehensive Meta-Analysis); random, observed+imputed values
