# Supplementary figures and images for: The impact of mechanical devices for lifting and transferring of patients on low back pain and musculoskeletal injuries in health care personnel—A systematic review and meta‐analysis
Source: J Occup Health. 2023 Sep 15;65(1):e12423. doi: 10.1002/1348-9585.12423 (PMC10502824; doi:10.1002/1348-9585.12423)

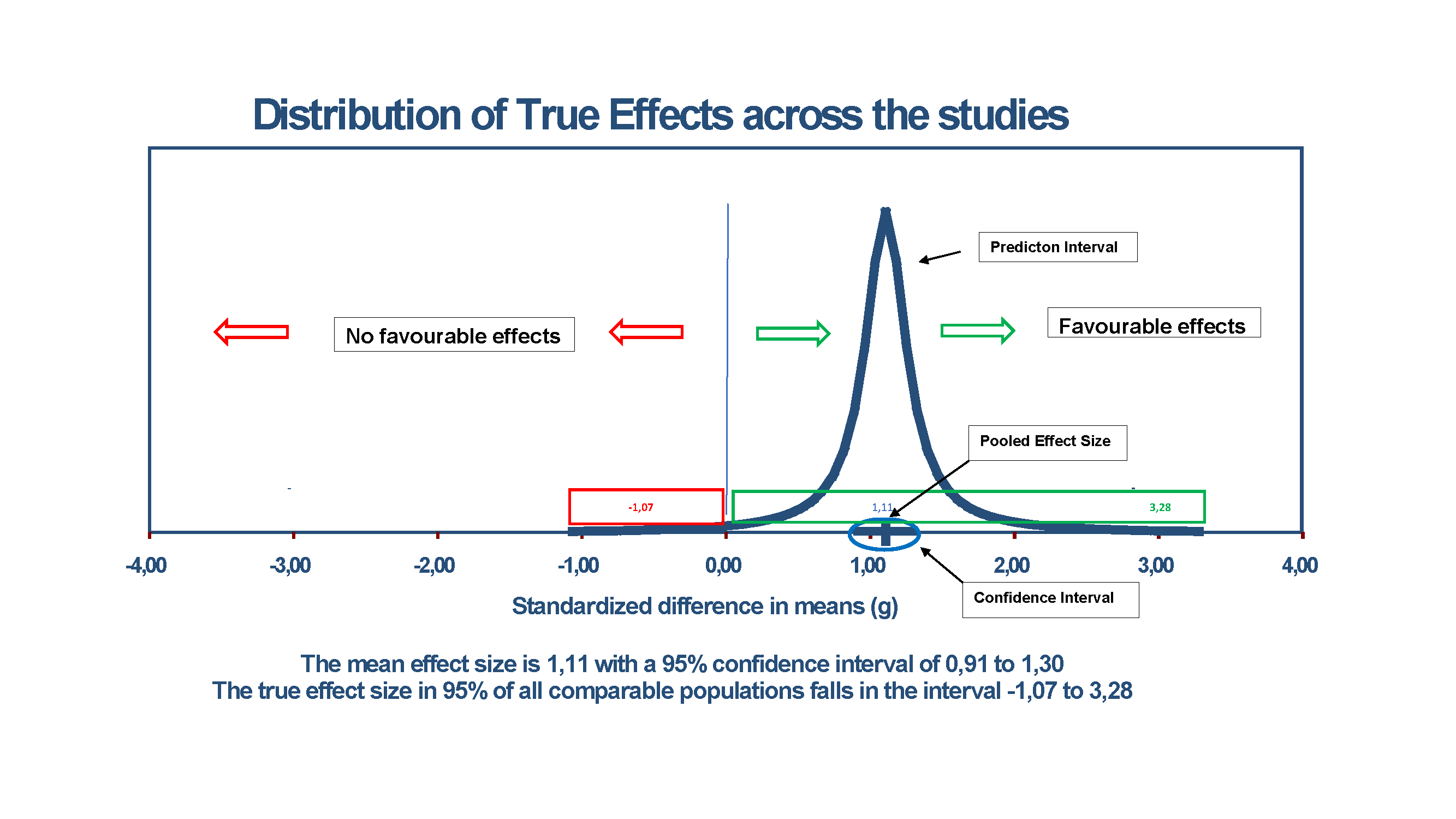

Supplement: Supplementary file 6 — Appendix F. [file JOH2-65-e12423-s004.zip › Appendix F_Figure A1_MSI Rate_Prediction Interval_26.03.2023.tiff]

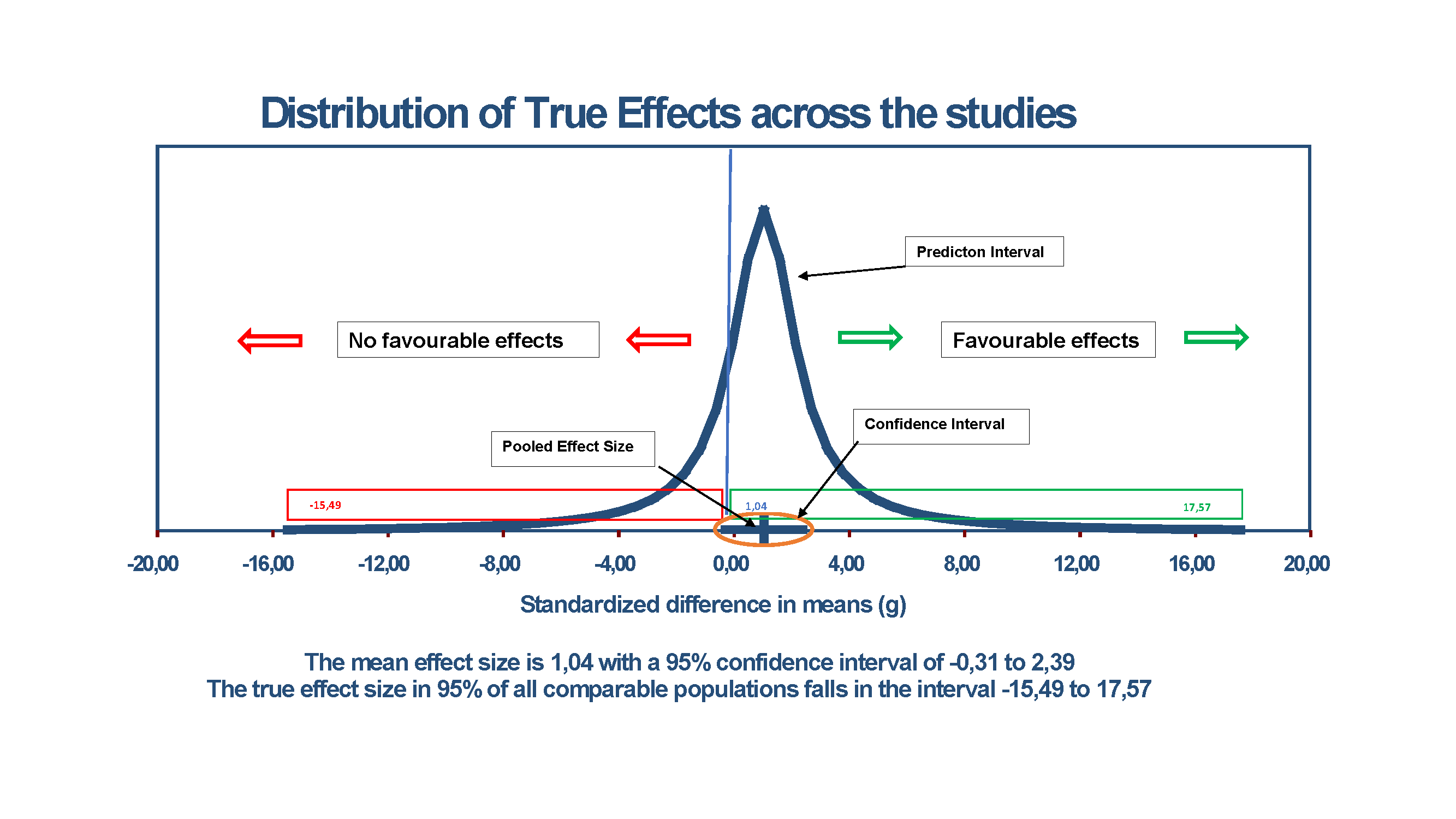

Supplement: Supplementary file 6 — Appendix F. [file JOH2-65-e12423-s004.zip › Appendix F_Figure A3_Peak Compr_Prediction Interval_26.03.2023.tiff]

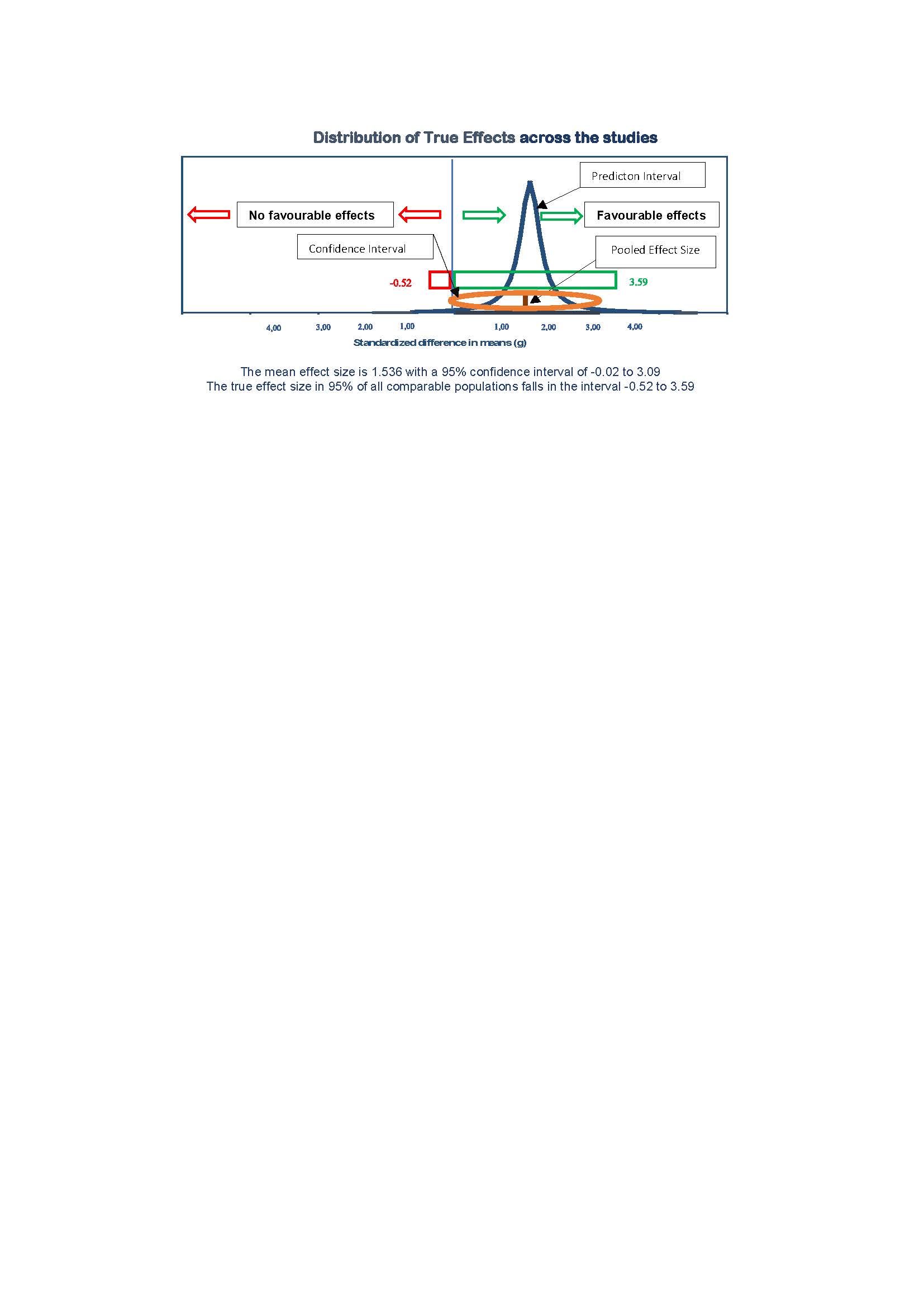

Supplement: Supplementary file 6 — Appendix F. [file JOH2-65-e12423-s004.zip › rev_Appendix F_Fig A2_LBP_Prediction Interval_27.07.2023.jpg]

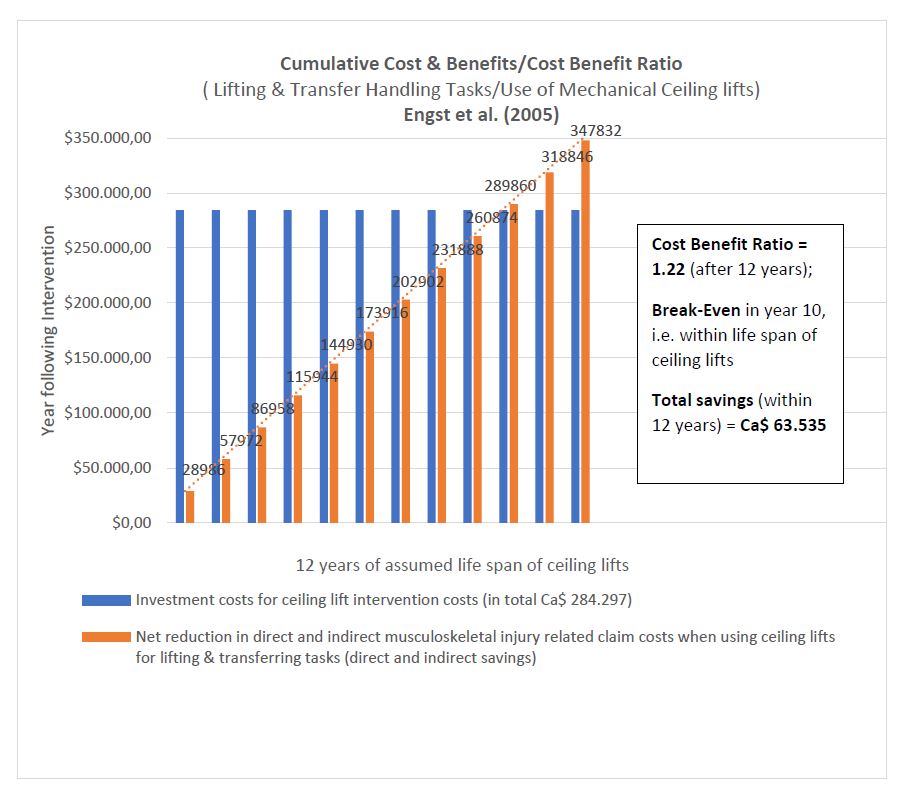

Supplement: Supplementary file 8 — Appendix H. [file JOH2-65-e12423-s002.zip › Appendix H_Figure_CBA_Engst_26.03.2023.JPG]

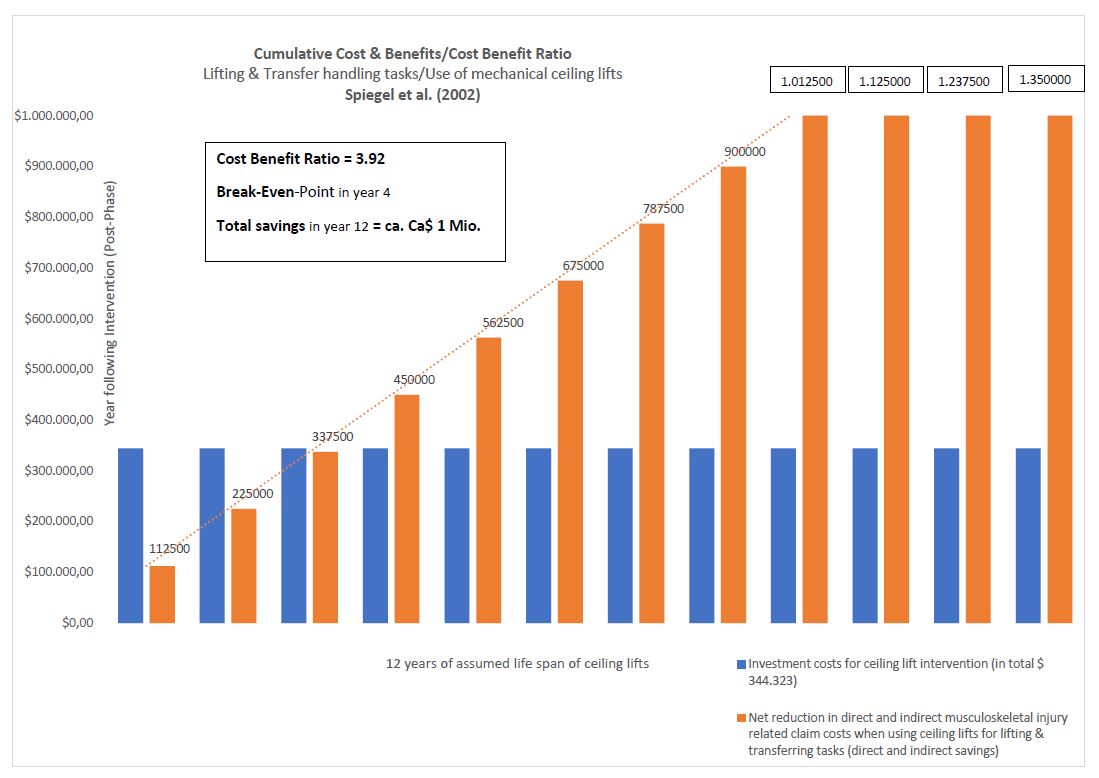

Supplement: Supplementary file 8 — Appendix H. [file JOH2-65-e12423-s002.zip › Appendix H_Figure_CBA_Spiegel_26.03.2023.JPG]
