# Supplementary material for: The impact of mechanical devices for lifting and transferring of patients on low back pain and musculoskeletal injuries in health care personnel—A systematic review and meta‐analysis
Source: J Occup Health. 2023 Sep 15;65(1):e12423. doi: 10.1002/1348-9585.12423 (PMC10502824; doi:10.1002/1348-9585.12423)
Supplement: Supplementary file 7 — Appendix G. [file JOH2-65-e12423-s006.zip › Appendix G_Table A4_RoB_Yassi et al_2001_26.03.2023.docx]

**Appendix F.** **Table A3.**  **Cochrane RoB 2.0** – *Risk of Bias Assessment Tool for randomized controlled studies (****Yassi et al., 2001****)*

Revised Cochrane risk-of-bias tool for randomized trials (RoB 2) TEMPLATE FOR COMPLETION

Edited by Julian PT Higgins, Jelena Savović, Matthew J Page, Jonathan AC Sterne on behalf of the RoB2 Development Group

# Version of 22 August 2019

The development of the RoB 2 tool was supported by the MRC Network of Hubs for Trials Methodology Research (MR/L004933/2- N61), with the support of the host MRC ConDuCT-II Hub (Collaboration and innovation for Difficult and Complex randomised controlled Trials In Invasive procedures - MR/K025643/1), by MRC research grant MR/M025209/1, and by a grant from The Cochrane Collaboration.


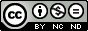


This work is licensed under a [Creative Commons Attribution-NonCommercial-NoDerivatives 4.0 International License](http://creativecommons.org/licenses/by-nc-nd/4.0/).

**Study details**

# Yassi A, Cooper JE, Tate RB et al. (2001). A randomized Controlled Trial to Prevent Patient Lift and Transfer Injuries of Health Care Workers, Spine 26 (16): 1739-1746.

# Workers, Spine 26 (16): 1739-1746.

# Reference

**Study design**

X Individually-randomized parallel-group trial

- Cluster-randomized parallel-group trial
- Individually randomized cross-over (or other matched) trial

# For the purposes of this assessment, the interventions being compared are defined as

1. “Safe lifting program (**Arm B**) and
2. “No strenuous lifting program (**Arm C**)

Experimental

Comparator:

1. Self-rated 7-day prevalence of LBP over time (baseline/6 m/12 m); 2. Musculoskeletal injuries (raw No and rate) during study year and previous year by service area and study arm.

“Usual HSC practice” (**Arm A**)

# Specify which outcome is being assessed for risk of bias

**Specify the numerical result being assessed.** In case of multiple alternative analyses being presented, specify the numeric result (e.g. RR = 1.52 (95% CI

Outcome 1. see Table 4

Outcome 2. see Table 8

0.83 to 2.77) and/or a reference (e.g. to a table, figure or paragraph) that uniquely defines the result being assessed.

# Is the review team’s aim for this result…?

X to assess the effect of *assignment to intervention* (the ‘intention-to-treat’ effect)

- to assess the effect of *adhering to intervention* (the ‘per-protocol’ effect)

**If the aim is to assess the effect of *adhering to intervention***, select the deviations from intended intervention that should be addressed (at least one must be checked):

□

□

□

occurrence of non-protocol interventions

failures in implementing the intervention that could have affected the outcome non-adherence to their assigned intervention by trial participants

**Which of the following sources were obtained to help inform the risk-of-bias assessment? (tick as many as apply)**

X Journal article(s) with results of the trial

- Trial protocol
- Statistical analysis plan (SAP)
- Non-commercial trial registry record (e.g. ClinicalTrials.gov record)
- Company-owned trial registry record (e.g. GSK Clinical Study Register record)
- “Grey literature” (e.g. unpublished thesis)
- Conference abstract(s) about the trial
- Regulatory document (e.g. Clinical Study Report, Drug Approval Package)
- Research ethics application
- Grant database summary (e.g. NIH RePORTER or Research Councils UK Gateway to Research)
- Personal communication with trialist
- Personal communication with the sponsor

Risk of bias assessment

Responses underlined in green are potential markers for low risk of bias, and responses in red are potential markers for a risk of bias. Where questions relate only to sign posts to other questions, no formatting is used.

**Domain 1: Risk of bias arising from the randomization process**

| **Signalling questions** | **Comments** | **Response options** |
| --- | --- | --- |
| **1.1 Was the allocation sequence random?** | Information limited to the comment that “each of the three wards within a service area was then randomly assigned to one arm of the study”  No information provided | PY |
| **1.2 Was the allocation sequence concealed until participants were enrolled and assigned to interventions?** |  | PY |
| **1.3 Did baseline differences between intervention groups suggest a problem with the randomization process?** | No information provided. | PN |
| **Risk-of-bias judgement** |  | Low |
| Optional: What is the predicted direction of bias arising from the randomization process? |  | NA |

Domain 2: Risk of bias due to deviations from the intended interventions (*effect of assignment to intervention*)

| **Signalling questions** | **Comments** | **Response options** |
| --- | --- | --- |
| **2.1. Were participants aware of their assigned intervention during the trial?** |  | PN |
| **2.2. Were carers and people delivering the interventions aware of participants'**  **assigned intervention during the trial?** |  | PY |
| **2.3. If Y/PY/NI to 2.1 or 2.2: Were there deviations from the intended intervention that arose because of the trial context?** |  | PN |
| **2.4 If Y/PY to 2.3: Were these deviations likely to have affected the outcome?** |  | PY |
| **2.5. If Y/PY/NI to 2.4: Were these deviations from intended intervention balanced between groups?** |  | PY |
| **2.6 Was an appropriate analysis used to estimate the effect of assignment to intervention?** |  | PY |
| **2.7 If N/PN/NI to 2.6: Was there potential for a substantial impact (on the result) of the failure to analyse participants in the group to which they were randomized?** |  | PN |
| **Risk-of-bias judgement** |  | Some concerns |
| Optional: What is the predicted direction of bias due to deviations from intended interventions? |  | NA |

Domain 2: Risk of bias due to deviations from the intended interventions (*effect of adhering to intervention*)

| **Signalling questions** | **Comments** | **Response options** |
| --- | --- | --- |
| **2.1. Were participants aware of their assigned intervention during the trial?** |  | PY |
| **2.2. Were carers and people delivering the interventions aware of participants' assigned intervention during the trial?** |  | NI |
| **2.3. [If applicable:] If Y/PY/NI to 2.1 or 2.2: Were important non-protocol interventions balanced across intervention groups?** |  | NI |
| **2.4. [If applicable:] Were there failures in implementing the intervention that could have affected the outcome?** |  | NI |
| **2.5. [If applicable:] Was there non- adherence to the assigned intervention regimen that could have affected participants’ outcomes?** |  | PN |
| **2.6. If N/PN/NI to 2.3, or Y/PY/NI to 2.4 or 2.5: Was an appropriate analysis used to estimate the effect of adhering to the intervention?** |  | PY |
| **Risk-of-bias judgement** |  | Some concerns |
| Optional: What is the predicted direction of bias due to deviations from intended interventions? |  | NA |

Domain 3: Missing outcome data

| **Signalling questions** | **Comments** | **Response options** |
| --- | --- | --- |
| **3.1 Were data for this outcome available for all, or nearly all, participants randomized?** |  | PN |
| **3.2 If N/PN/NI to 3.1: Is there evidence that the result was not biased by missing outcome data?** |  | PN |
| **3.3 If N/PN to 3.2: Could missingness in the outcome depend on its true value?** |  | PY |
| **3.4 If Y/PY/NI to 3.3: Is it likely that missingness in the outcome depended on its true value?** |  | PY |
| **Risk-of-bias judgement** |  | High |
| Optional: What is the predicted direction of bias due to missing outcome data? |  | NA |

Domain 4: Risk of bias in measurement of the outcome

| **Signalling questions** | **Comments** | **Response options** |
| --- | --- | --- |
| **4.1 Was the method of measuring the outcome inappropriate?** |  | PN |
| **4.2 Could measurement or ascertainment of the outcome have differed between intervention groups?** |  | PN |
| **4.3 If N/PN/NI to 4.1 and 4.2: Were outcome assessors aware of the intervention received by study participants?** |  | NI |
| **4.4 If Y/PY/NI to 4.3: Could assessment of the outcome have been influenced by knowledge of intervention received?** |  | PN |
| **4.5 If Y/PY/NI to 4.4: Is it likely that assessment of the outcome was influenced by knowledge of intervention received?** |  | NA |
| **Risk-of-bias judgement** |  | Low |
| Optional: What is the predicted direction of bias in measurement of the outcome? |  | NA |

Domain 5: Risk of bias in selection of the reported result

| **Signalling questions** | **Comments** | **Response options** |
| --- | --- | --- |
| **5.1 Were the data that produced this result analysed in accordance with a pre-specified analysis plan that was finalized before unblinded outcome data were available for analysis?** |  | PY |
| **Is the numerical result being assessed likely to have been selected, on the basis of the results, from...**  **5.2. multiple eligible outcome**  **measurements (e.g. scales, definitions, time points) within the outcome domain?** |  |  |
|  |  | PN |
| **5.3 ... multiple eligible analyses of the data?** |  | PN |
| **Risk-of-bias judgement** |  | Low |
| Optional: What is the predicted direction of bias due to selection of the reported result? |  | NA / |

Overall risk of bias

| **Risk-of-bias judgement** |  | High |
| --- | --- | --- |
| Optional: What is the overall predicted |  | NA |
| direction of bias for this outcome? |  |  |
|  |  |  |
|  |  |  |
|  |  |  |


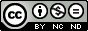


This work is licensed under a [Creative Commons Attribution-NonCommercial-NoDerivatives 4.0 International License](http://creativecommons.org/licenses/by-nc-nd/4.0/).
