# Supplementary material for: The impact of mechanical devices for lifting and transferring of patients on low back pain and musculoskeletal injuries in health care personnel—A systematic review and meta‐analysis
Source: J Occup Health. 2023 Sep 15;65(1):e12423. doi: 10.1002/1348-9585.12423 (PMC10502824; doi:10.1002/1348-9585.12423)
Supplement: Supplementary file 7 — Appendix G. [file JOH2-65-e12423-s006.zip › Appendix G_Tables A1-A3_EPOC+MINORS_26.03.2023.docx]

**APPENDIX F:** Quality Assessment of studies - individual and overall ratings

**Table A1:** **EPOC** *Risk of Bias* *Assessment Tool* *for* ***Before-After-Studies*** *with separate control group*

|  | **EPOC criteria and scores* applied to Before-After studies with separate control group included in the review** | | | | | | | | | |
| --- | --- | --- | --- | --- | --- | --- | --- | --- | --- | --- |
| **Study** (Year of publication) | Adequate sequence generation | Allocation Concealment | Blinding | Incomplete outcome data | Free of selective reporting | Free of other bias? | Baseline outcomes similar | Free of contamination | Baseline characteristics similar | Overall score* |
| **Fragala et al.** (2012) | **0** | **0** | **1** | **1** | **2** | **0** | **0** | **2** | **0** | High Risk 6/18 |
| **Knibbe et al.** (1999) | **0** | **0** | **0** | **1** | **2** | **1** | **0** | **2** | **1** | High Risk  7/18 |
| **Miller et al.** (2006) | **0** | **0** | **1** | **0** | **2** | **1** | **0** | **2** | **2** | High Risk  8/18 |
| **Owen et al.** (2002) | **0** | **2** | **0** | **2** | **2** | **2** | **2** | **2** | **1** | Low Risk  13/18 |
|  | ***EPOC** **RoB Scores:** **0**=High Risk **1**=Unclear Risk **2**= Low Risk  Evaluation of overall scores: 12 to 18 points = Low Risk less than 12 points = High Risk | | | | | | | | | |

**Table A2:** **EPOC** *Risk of Bias Assessment Tool for* ***Interrupted Time Series****-Studies*

|  | **EPOC criteria and scores* applied to Interrupted Time Series-Studies included in the review** | | | | | | | | |
| --- | --- | --- | --- | --- | --- | --- | --- | --- | --- |
| **Study** (Year of publication) | Adequate sequence generation | Allocation Concealment | Blinding | Incomplete outcome data | Free of selective reporting | Free of other bias? | Baseline outcomes similar | Free of contamination | Overall score* |
| **Alamgir et al.** (2008) | **0** | **2** | **2** | **2** | **1** | **1** | **2** | **0** | Low Risk  10/16 |
| **Anyan et al.** (2013) | **0** | **2** | **2** | **2** | **1** | **1** | **2** | **1** | Low Risk  11/16 |
| **Collins et al.** (2004) | **2** | **2** | **2** | **2** | **1** | **0** | **2** | **2** | Low Risk  13/16 |
| **Chhokar et al.** (2005) | **1** | **1** | **1** | **2** | **1** | **1** | **2** | **0** | High Risk  13/16 |
| **Engst et al.** (2005) | **1** | **2** | **2** | **2** | **1** | **1** | **2** | **0** | Low Risk  11/16 |
| **Ronald et al.** (2002) | **1** | **1** | **2** | **2** | **1** | **1** | **2** | **2** | Low Risk  12/16 |
| **Spiegel et al. (**2002) | **1** | **1** | **2** | **2** | **1** | **1** | **2** | **1** | Low Risk  11/16 |
|  | ***EPOC** **RoB Scores:** **0**=High Risk **1**=Unclear Risk **2**= Low Risk  Evaluation of overall scores: 10 to 16 points = Low Risk 0 to 9 points = High Risk | | | | | | | | |
